# Efficacy and safety of particle therapy for inoperable stage II-III non-small cell lung cancer: a systematic review and meta-analysis

**DOI:** 10.1186/s13014-023-02264-x

**Published:** 2023-05-22

**Authors:** Yanliang Chen, Hongtao Luo, Ruifeng Liu, Mingyu Tan, Qian Wang, Xun Wu, Tianqi Du, Zhiqiang Liu, Shilong Sun, Qiuning Zhang, Xiaohu Wang

**Affiliations:** 1 Institute of Modern Physics, Chinese Academy of Sciences, Lanzhou, 730000 Gansu Province China; 2 The First School of Clinical Medicine, Lanzhou University, Lanzhou, China; 3 Department of Postgraduate, University of Chinese Academy of Sciences, Beijing, China; 4 Heavy Ion Therapy Center, Lanzhou Heavy Ions Hospital, Lanzhou, China

**Keywords:** Non-small cell lung cancer (NSCLC), Particle therapy, Carbon-ion radiotherapy (CIRT), Proton beam therapy (PBT), Meta-analysis

## Abstract

**Background and purpose:**

Particle therapy, mainly including carbon-ion radiotherapy (CIRT) and proton beam therapy (PBT), has dose distribution advantages compared to photon radiotherapy. It has been widely reported as a promising treatment method for early non-small cell lung cancer (NSCLC). However, its application in locally advanced non-small cell lung cancer (LA-NSCLC) is relatively rare, and its efficacy and safety are inconclusive. This study aimed to provide systematic evidence for evaluating the efficacy and safety of particle therapy for inoperable LA-NSCLC.

**Methods:**

To retrieve published literature, a systematic search was conducted in PubMed, Web of Science, Embase, and Cochrane Library until September 4, 2022. The primary endpoints were local control (LC) rate, overall survival (OS) rate, and progression-free survival (PFS) rate at 2 and 5 years. The secondary endpoint was treatment-related toxicity. The pooled clinical outcomes and 95% confidence intervals (CIs) were calculated by using STATA 15.1.

**Results:**

Nineteen eligible studies with a total sample size of 851 patients were included. The pooled data demonstrated that the OS, PFS, and LC rates at 2 years of LA-NSCLC treated by particle therapy were 61.3% (95% CI = 54.7-68.7%), 37.9% (95% CI = 33.8-42.6%) and 82.2% (95% CI = 78.7-85.9%), respectively. The pooled 5-year OS, PFS, and LC rates were 41.3% (95% CI = 27.1-63.1%), 25.3% (95% CI = 16.3-39.4%), and 61.5% (95% CI = 50.7-74.6%), respectively. Subgroup analysis stratified by treatment type showed that the concurrent chemoradiotherapy (CCRT, PBT combined with concurrent chemotherapy) group had better survival benefits than the PBT and CIRT groups. The incidence rates of grade 3/4 esophagitis, dermatitis, and pneumonia in LA-NSCLC patients after particle therapy were 2.6% (95% CI = 0.4-6.0%), 2.6% (95% CI = 0.5-5.7%) and 3.4% (95% CI = 1.4-6.0%), respectively.

**Conclusions:**

Particle therapy demonstrated promising efficacy and acceptable toxicity in LA-NSCLC patients.

**Supplementary Information:**

The online version contains supplementary material available at 10.1186/s13014-023-02264-x.

## Background

Lung cancer ranks second in cancer incidence and first in terms of cancer mortality around the world, with non-small cell lung cancer (NSCLC) accounting for 80-85% of the lung cancer diagnoses [1, 2]. Nevertheless, there is a subset of patients with NSCLC who are not suitable candidates for surgical resection due to various reasons such as locally advanced or metastatic disease, advanced age, severe underlying disease, and refusal to undergo surgical intervention [3]. Traditionally, the standard therapeutic regimen for the management of locally advanced non-small cell lung cancer (LA-NSCLC) has been the application of thoracic radiotherapy in conjunction with cisplatin-based chemotherapy [4], with 5-year overall survival rates ranging between 16 and 32% for inoperable stage III NSCLC [5–7]. The landscape of treatment for LA-NSCLC has been transformed in recent times following the outcomes of the PACIFIC trial, which introduced consolidation immunotherapy subsequent to definitive chemo-radiotherapy [8]. Notwithstanding the unparalleled findings displaying a median overall survival (OS) of 47.5 months, which stands as the highest ever recorded for unresectable LA-NSCLC, further advancements are still possible. Furthermore, certain patients may not meet the eligibility criteria for Durvalumab due to factors such as comorbidities, performance status (PS), or low PD-L1 expression.

The positive correlation between escalated radiotherapy dose and improved tumor control probability has been established [9–11], yet the translation of this association into clinical outcomes has not been successful due to the increased incidence of treatment-related toxicities in organs at risk (OARs, lung, esophagus, heart, etc.). The RTOG (Radiation Therapy Oncology Group) 0617 trial serves as a prime example of this dilemma [12]. All of this tells us that mitigating treatment toxicity is crucial in the radiotherapy of LA-NSCLC.

Over the past two decades, particle therapy, mainly comprising carbon-ion radiotherapy (CIRT) and proton beam therapy (PBT), has been utilized for the treatment of various cancers, including lung cancer, and has shown encouraging clinical outcomes and acceptable toxicity [13–16]. It was estimated that approximately 13% of patients receiving curative radiotherapy might benefit from PBT [17]. Compared to photon radiotherapy, particle therapy has a better dose distribution [18, 19], which is reflected in when a similar or higher dose is given to tumor tissue, the radiation dose exposed to normal tissue is lower or the same [20]. Patients with LA-NSCLC present unique challenges in treatment due to their larger irradiation field, higher risk of severe treatment toxicity, and greater susceptibility to local recurrence post-treatment compared to those with early-stage NSCLC. As a result, particle therapy is a potentially more suitable treatment option for LA-NSCLC patients.

In recent years, there has been an increasing focus on the efficacy and safety of particle therapy for NSCLC. However, most of the research has been concentrated in the field of early-stage NSCLC, with relatively few studies focusing on the more significant concern of LA-NSCLC. The vast majority of these studies are observational [21], with the results showing promise [22–26]. To guide clinical practice, we have decided to conduct an evidence-based meta-analysis to evaluate the safety and efficacy of particle therapy in treating LA-NSCLC.

## Methods

A prospective registration for the protocol was made in PROSPERO (registration number: CRD42022322132). The Preferred Reporting Items for Systematic Reviews and Meta-Analyses (PRISMA) guideline was used to report our findings [27].

### Search strategy

To retrieve published literature, a systematic search was conducted in PubMed, Web of Science, Embase, and Cochrane Library until September 4, 2022. This meta-analysis was not language-restricted. Search terms, comprising free text words and MESH terms, related to charged particle treatment of NSCLC were utilized in various combinations and plural forms to conduct an exhaustive literature search. The main search items are as follows: particle*, heavy ion*, carbon, C-ion, proton*, Carcinoma, Non-Small-Cell Lung, and Non-Small-Cell Lung Carcinomas. The present study did not impose any restrictions on the publication year, language, or study design in order to conduct an exhaustive search for relevant research papers. Additionally, a manual review of references from selected papers was conducted to identify additional research papers that might have been overlooked. The detailed search strategy for each database is shown in the Supplementary.

### Inclusion and exclusion criteria

The inclusion criteria were as follows: All types of primary studies published in English that reported the outcomes of using particle therapy as a definitive treatment (with or without concurrent chemotherapy) in patients with an inoperable stage II-III NSCLC were considered for inclusion, except case reports. Neither the publication date nor the population or design of the study were restricted. To ensure the accuracy and quality of the data analyzed in the study, several exclusion criteria were applied. These criteria were: (i) exclusion of duplicate reports, with only the most recent or parent study being included; (ii) exclusion of abstracts for which the complete text was not available; (iii) exclusion of studies reporting cases of NSCLC concurrent with other malignant neoplasms; (iv) exclusion of studies reporting only protocols; and (v) exclusion of studies from which data could not be extracted.

### Data extraction and endpoints

The relevant data were extracted from the studies independently by two investigators (Luo Hongtao and Liu Ruifeng). For each study, the following characteristics were recorded: first author, publication year, study design, number of patients, the mean age of patients, institution, treatment protocol, dose and fractionation regimens, follow-up period, the proportion of medically inoperable patients, and treatment-related toxicity. Any discrepancy was resolved by consensus with the third party (Zhang Qiuning).

### Quality assessment

Only case-control and case series studies have been reported on this subject; no controlled randomized trials have been conducted. Therefore, the quality evaluation is based on the non-randomized research methodology index (MINORS) [28], which includes eight items applicable to non-comparative and comparative studies and four additional criteria applicable to comparative studies. Du Tianqi and Tan Mingyu, two authors, independently assessed the quality of each study. If they disagree, Wang Xiaohu, the third author, is asked to decide.

### Statistical analysis

The clinical outcomes of interest include local control (LC), overall survival (OS), progression-free survival (PFS) at 2 and 5 years, and treatment-related toxicity. Some studies only reported efficacy indicators, such as 2-year OS, without reporting their 95% confidence intervals. Therefore, missing data on the 95% CI of the efficacy indicators in the study were handled using multiple imputations. M datasets are generated by Rubin’s multiple imputation, and each dataset replaces the missing value with a reasonable value, which represents the uncertainty of the correct imputation value [29]. In the multiple imputation method, the imputation variables are obtained from the density function created by the regression model [30], which employs other variables (i.e., covariates and outcomes) to forecast the missing value of a specific variable. This method properly reflects the uncertainty caused by missing values and results in statistically valid inferences. We created ten datasets (m = 10).

Meta-analysis was conducted to pool OS rate, LC rate, and PFS rate at 2 and 5 years, as well as the incidence of common adverse events. An assessment of heterogeneity included the chi-square test and I-squared test value (values < 25% indicate low heterogeneity; 25–75% moderate heterogeneity; and > 75% considerable heterogeneity). A p-value less than 0.1 was defined as a statistically significant difference. When I2 was greater than 25%, a random effects model was utilized to combine the incidence rate and its 95% confidence interval. Otherwise, a fixed effect model would be used.

Subgroup analysis was performed based on the TNM stage and type of particle therapy to assess the impact of these factors on the integrated results and try to explain the source of heterogeneity. The sensitivity analysis conducted in this study included two parts. Firstly, a complete-case analysis was performed to evaluate the impact of multiple imputation. Secondly, we carried out a meta-analysis by excluding one cohort at a time to examine the effect of each cohort on our pooled estimates.

Multiple imputation was performed using R statistical software (version 4.2.0, “mice”package). SPSS version 25.0 and STATA version 15.1 are used to manage and analyze data.

## Results

### Search results

**Fig. 1:**
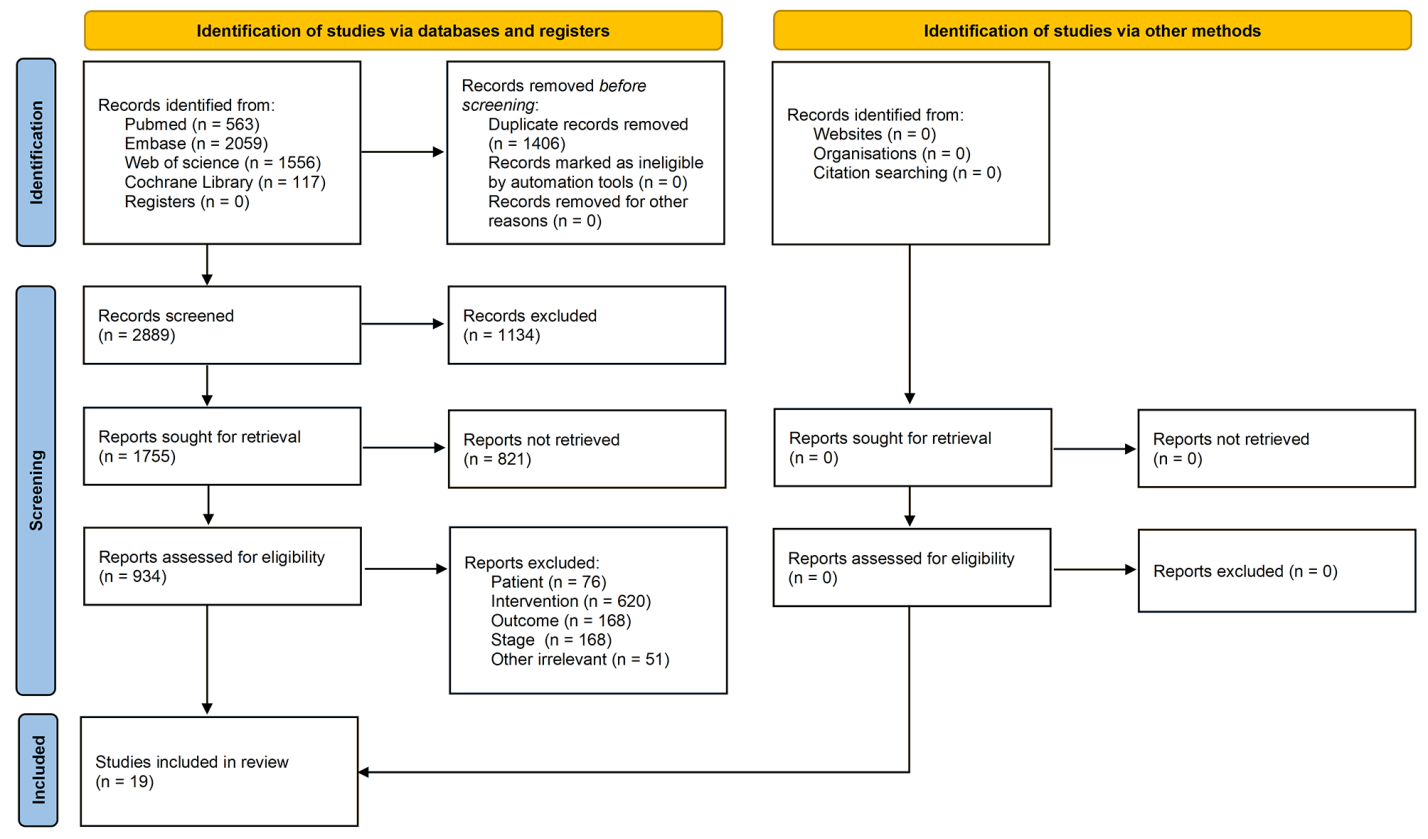
Flow chart of study selection

A total of 4295 studies were found in our initial search. There were 2889 studies left after excluding 1406 repetitive studies. 2311 studies were excluded because they were considered not to meet the inclusion criteria after screening the titles and abstracts. 508 reviews and 51 other unrelated articles were eliminated by reading the full text. Eventually, this meta-analysis included 19 studies involving 851 patients with LA-NSCLC. We outline the study selection process for review using the PRISMA flowchart (Fig. 1).

### Study characteristics

Eight of the included studies were phase I / II clinical trials, and the rest were almost all case-series studies, except for one case-control study comparing the efficacy of proton therapy and photon therapy, in which we extracted the data as reported on proton therapy. There were three studies with 119 patients receiving PBT [31–33], 11 studies with 452 patients receiving CCRT [24, 34–43] (concurrent chemoradiotherapy, both PBT and concurrent chemotherapy), and four studies with 235 patients receiving CIRT [25, 26, 44, 45]. In another study, most patients received PBT, and others received CIRT [46]. The 2-year OS rate, PFS rate, and LC rate were reported by 15 studies, 13 studies, and 11 studies, respectively. Regarding the 5-year survival data, OS rates and PFS rates were reported by five studies. According to the MINORS criteria, the median quality score for case series studies was 11 (range 10–12), and the only control study was 18. The complete quality assessment is available in Supplementary Table 1. The characteristics of 19 included studies are summarized in Table 1.

**Table 1:** Study and population characteristics for included studies

First author	Year	Study design	Sample size	Stage	Median/mean age (range)	Median follow-up (months)	Treatment type	Fraction dose (GyE)	Total dose (GyE)	EQD2	PS	Medically inoperable
Nakayama	2011	R	35	II-III	70 (47-85)	16.9 (NR)	PBT	2-3.3	72.6-83.6	67.1-91.3	0-2	88.6%
Oshiro	2012	R	57	III	72 (42-85)	16.2 (NR)	PBT	2-6.6	50-84.5	NC	0-2	NR
Iwata	2013	R	70	II	75 (57-92)	44 (4-103)	PBT or CIRT	2.7-13.2	52.8-80	77.7-102.1	0-2	57.1%
Oshiro	2014	II	15	III	60 (40-68)	21.7 (NR)	PBT+CC	2	74	74	0-1	NR
Hoppe	2015	II	14	III	65 (48-82)	30 (NR)	PBT+CC	2	12-80	12-80	0-1	100%
Nguyen	2015	P	134	II-III	69 (28-95)	55.2 (18-80.4)	PBT+CC	2	60-74.1	60-74.1	NR	NR
Hatayama	2015	R	27	III	72 (57-91)	15.4 (7.8-36.9)	PBT	2-3.2	66-86.4	NC	0-1	NR
Harada	2016	I	9	III	72 (56-74)	43 (NR)	PBT+CC	2	60-74	60-74	0-1	100%
Chang	2017	II	64	III	70 (37-78)	27.3 (2.7-111.5)	PBT+CC	2	74	74	NR	NR
Shirai	2017	R	23	II-III	78 (53-91)	25 (4-54)	CIRT	4-15	52.8-70.4	74.7-125.0	0-2	52.2%
Saitoh	2018	I	6	III	77 (64-80)	26 (4-43)	CIRT	4	64	74.7	0-1	100%
Hayashi	2018	R	141	II-III	75 (40-88)	29.3 (1.6-207.7)	CIRT	NR	54-76	NC	0-2	78.7%
Elhammali	2019	R	51	II-III	70 (43-83)	23.0 (0.9-60.1)	PBT+CC	NR	59.4-78.0	NC	NR	NR
Iwata	2020	II	47	III	65 (31-74)	37 (4-84)	PBT+CC	2	70	70	0-1	100%
Anzai	2020	R	65	III	73 (40-88)	27.6 (1.6-207.7)	CIRT	4-4.75	64-76	74.7-93.4	0-2	83%
Kim	2021	R	25	III	62 (56-75)	21.7 (16.8-26.8)	PBT+CC	2-2.2	59.4-74	60-74	0-2	NR
Ohnishi	2021	R	45	III	62 (39-79)	42.1 (6.4-127.0)	PBT+CC	2	74	74	0-1	NR
Contreras	2021	I	20	II-III	66 (52-89)	20.3 (1-38)	PBT+CC	3.5-4	52.5-60	59.1-70	0-2	NR
Hoppe	2022	I/II	28	II-III	70 (50-86)	31 (1-82)	PBT+CC	2.5-4	60	62.5-70.0	0-1	NR

Abbreviations: Gy, gray (for particle therapy gray equivalent (GyE) ); EQD2, biological equivalent dose for the tumor in 2 Gy fractions; P, prospective; R, retrospective; I, Phase I; II, Phase II; PBT, proton beam therapy; CIRT, carbon-ion radiotherapy; CC, concurrent chemotherapy; PS, Performance Status; NR, not reported; NC, not calculable

### Efficacy

**Fig. 2:**
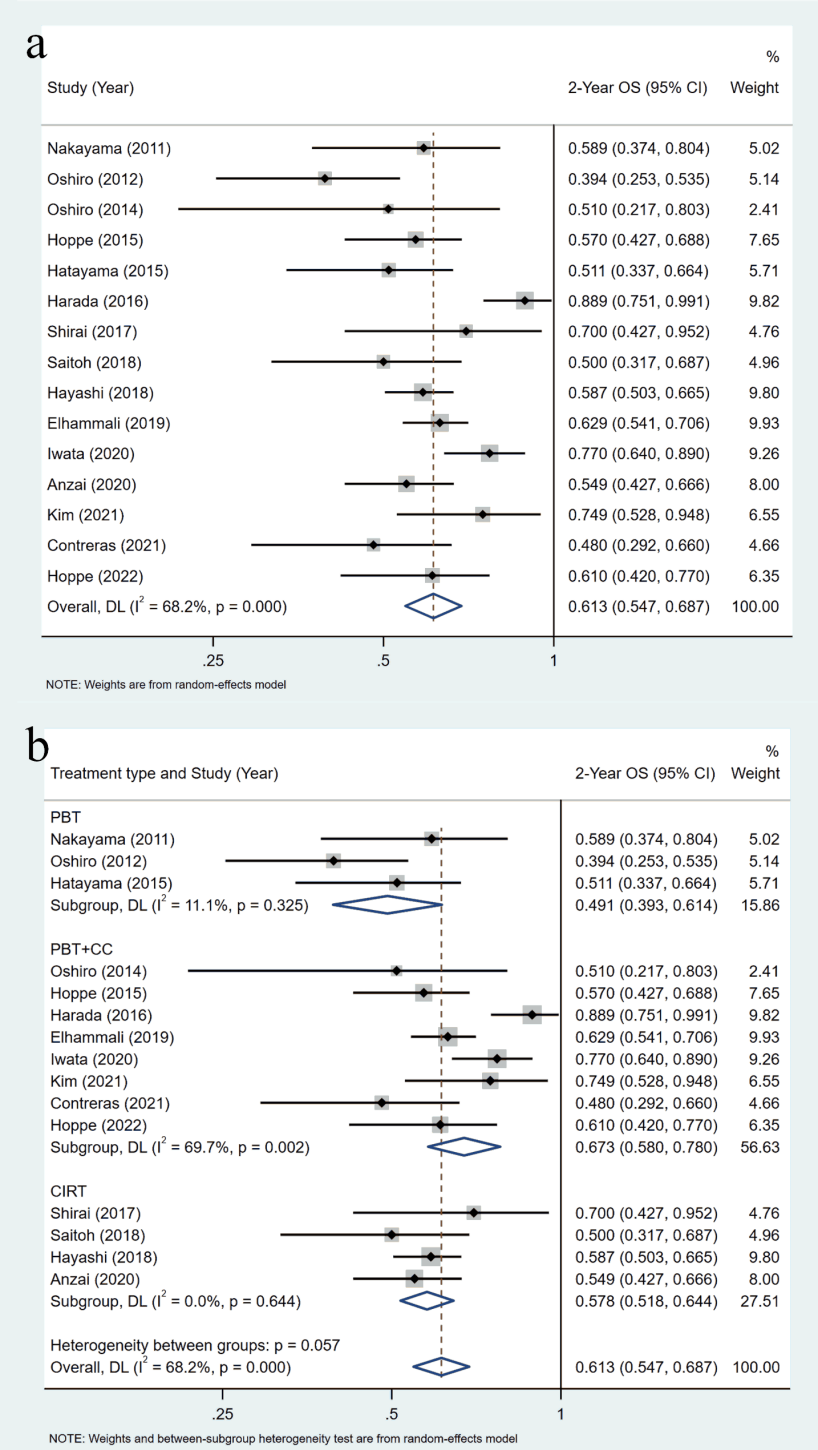
Meta-analysis of the 2-year overall survival rate (OS): (a) 2-year OS, overall; (b) 2-year OS, subgroup analysis stratified by treatment type. Abbreviations: PBT, proton beam therapy; CIRT, carbon-ion radiotherapy; CC, concurrent chemotherapy

There were 15 studies reported 2-year OS, which included 563 patients. The pooled 2-year OS rate of LA-NSCLC treated by particle therapy was 61.3% (95%CI = 54.7-68.7%), with high inter-study heterogeneity (I2 = 68.2%, p = 0.00) (Fig. 2a). Based on the treatment type subgroup, the 2-year OS of CCRT group was the highest (67.3%, 95%CI = 58.0-78.0%, I2 = 69.7%, p = 0.002), the 2-year OS of PBT group was the lowest (49.1%, 95%CI = 39.3-61.4%, I2 = 11.1%, p = 0.325), the 2-year OS of CIRT group was 57.8% (95%CI = 51.8-64.4%) and the heterogeneity level was low (I2 = 0.0%, p = 0.644) (Fig. 2b). The pooled 2-year OS rate of stage II-III group (61%, 95%CI = 56-66%) was the same with that of the stage II-III group (61%, 95%CI = 51-73%) (Supplementary Fig. S1a).

**Fig. 3:**
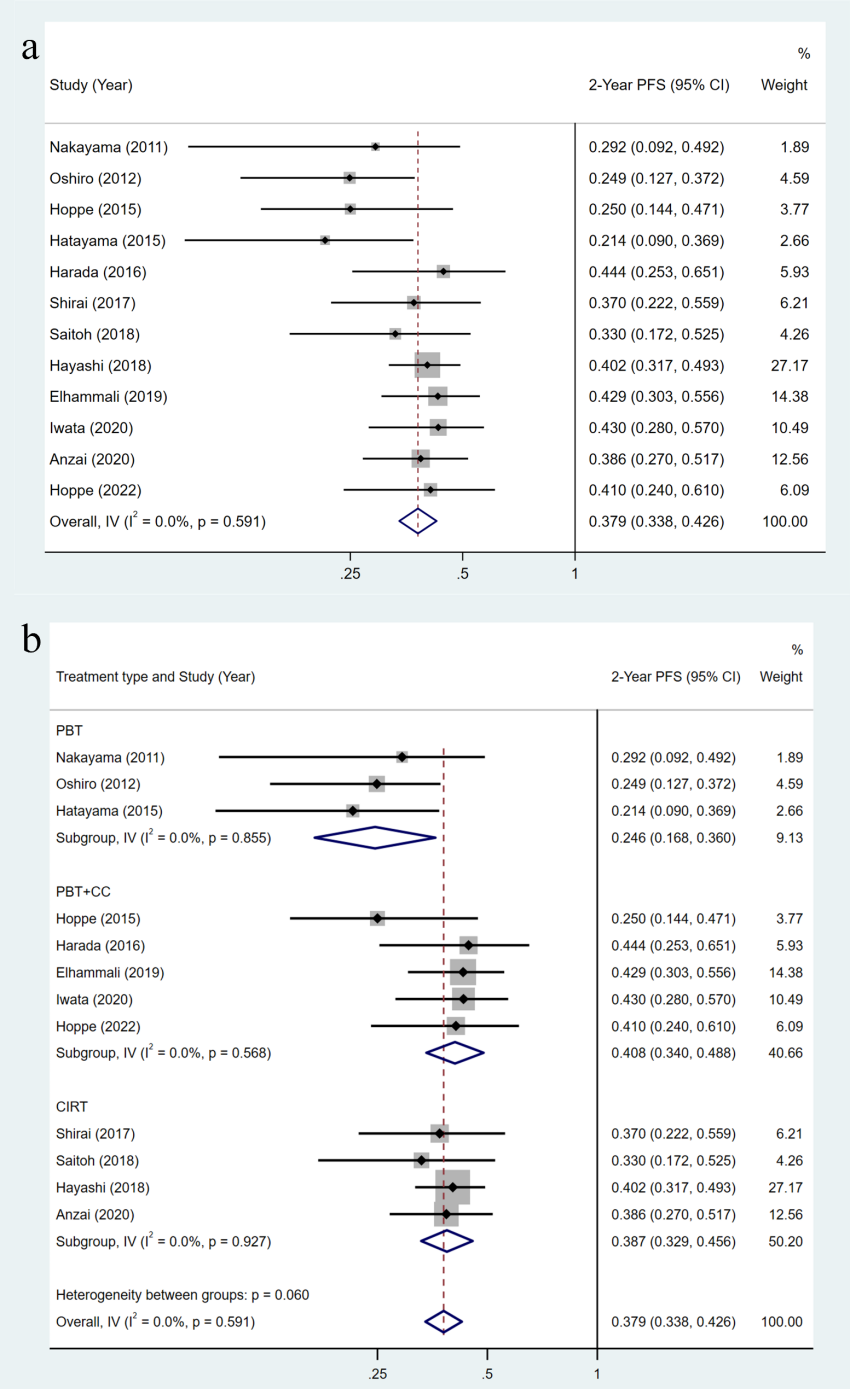
Meta-analysis of the 2-year progression-free survival (PFS): (a) 2-year PFS, overall; (b) 2-year PFS, subgroup analysis stratified by treatment type. Abbreviations: PBT, proton beam therapy; CIRT, carbon-ion radiotherapy; CC, concurrent chemotherapy

The pooled 2-year PFS of the 13 included studies included 518 patients was 37.9% (95%CI = 33.8-42.6%), with low inter-study heterogeneity (I2 = 0.0%, p = 0.591) (Fig. 3a). The CCRT group showed the highest pooled 2-year PFS of 40.8% (95%CI = 34.0-48.8%), followed by the CIRT group for 38.7% (95%CI = 32.9-45.6%) and finally the PBT group for 24.6% (95%CI = 16.8-36.0%) (Fig. 3b). The pooled 2-year PFS was 40.2% (95%CI = 34.4-46.8%) for the stage II-III group and 35.3% (95%CI = 29.7-42.0%) for the stage III group, with no statistical difference between the two subgroups (P = 0.278) (Supplementary Fig. S1b).

Data on 2-year LC rate to evaluate the effect of particle therapy were available in 11 studies, which included 505 patients, with a pooled value of 82.2% (95%CI = 78.7-85.9%, I2 = 15.2%, p = 0.299) (Supplementary Fig. S2a). 2-year LC in the PBT group, CIRT group, and CCRT group were 61.9%, 79.1%, and 85.0%, respectively (P = 0.013) (Supplementary Fig. S2b). 2-year LC of the stage II-III group was similar to that of the stage III group (81.6% VS 82.7%, P = 0.771) (Supplementary Fig. S2c).

**Fig. 4:**
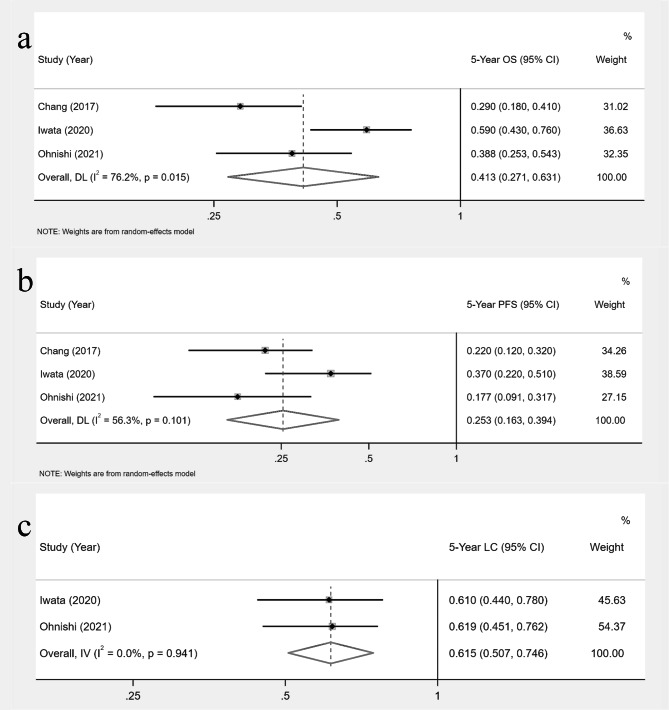
Meta-analysis of the overall survival rate (OS) (a), progression-free survival (PFS) (b), and local control (LC) (c) at 5 year

Only five studies reported the OS and PFS data of patients at 5 years after particle therapy, two of which did not report the 95% confidence interval of survival data. Due to limited data, multiple imputation is not feasible, so we combined three studies that reported complete data in which all patients were stage III and received CCRT treatment. The pooled 5-year OS and PFS of patients treated with CCRT were 41.3% (95%CI = 27.1-63.1%) and 25.3% (95%CI = 16.3-39.4%), respectively (Fig. 4a-b). Three studies reported the data of the 5-year LC rate. For the above reasons, we integrated two studies with complete data in which all patients were stage III and received CCRT treatment. The pooled 5-year LC of patients treated with CCRT was 61.5% (95%CI = 50.7-74.6%) (Fig. 4c).

### Safety

The present study evaluated the occurrence rates of common adverse events, such as esophagitis, pneumonitis, and dermatitis, in LA-NSCLC patients who underwent particle therapy. All adverse events (both acute toxicity and late toxicity) were classified as grade 2 or grade 3/4.

The incidences of grade 2 and grade 3/4 dermatitis were 19.8% (95%CI = 14.8-25.3%) and 2.6% (95%CI = 0.5-5.7%), respectively (Supplementary Fig. S3a-b). The subgroup analysis of treatment type showed that the incidences of grade 2/3/4 dermatitis in the CCRT group were higher than in the PBT group and CIRT group, similar to that of esophagitis. The incidences of grade 2 dermatitis in PBT, CIRT, and CCRT groups were 15.5%, 12.2%, and 26.0%, respectively, and the incidences of grade 3/4 dermatitis were 4.2%, 0.0%, and 5.1% respectively (Supplementary Fig. S4a-b). Subgroup analysis stratified by stage showed that there was no difference in the incidences of dermatitis (grade 2/3/4) among groups (Supplementary Fig. S5a-b).

**Fig. 5:**
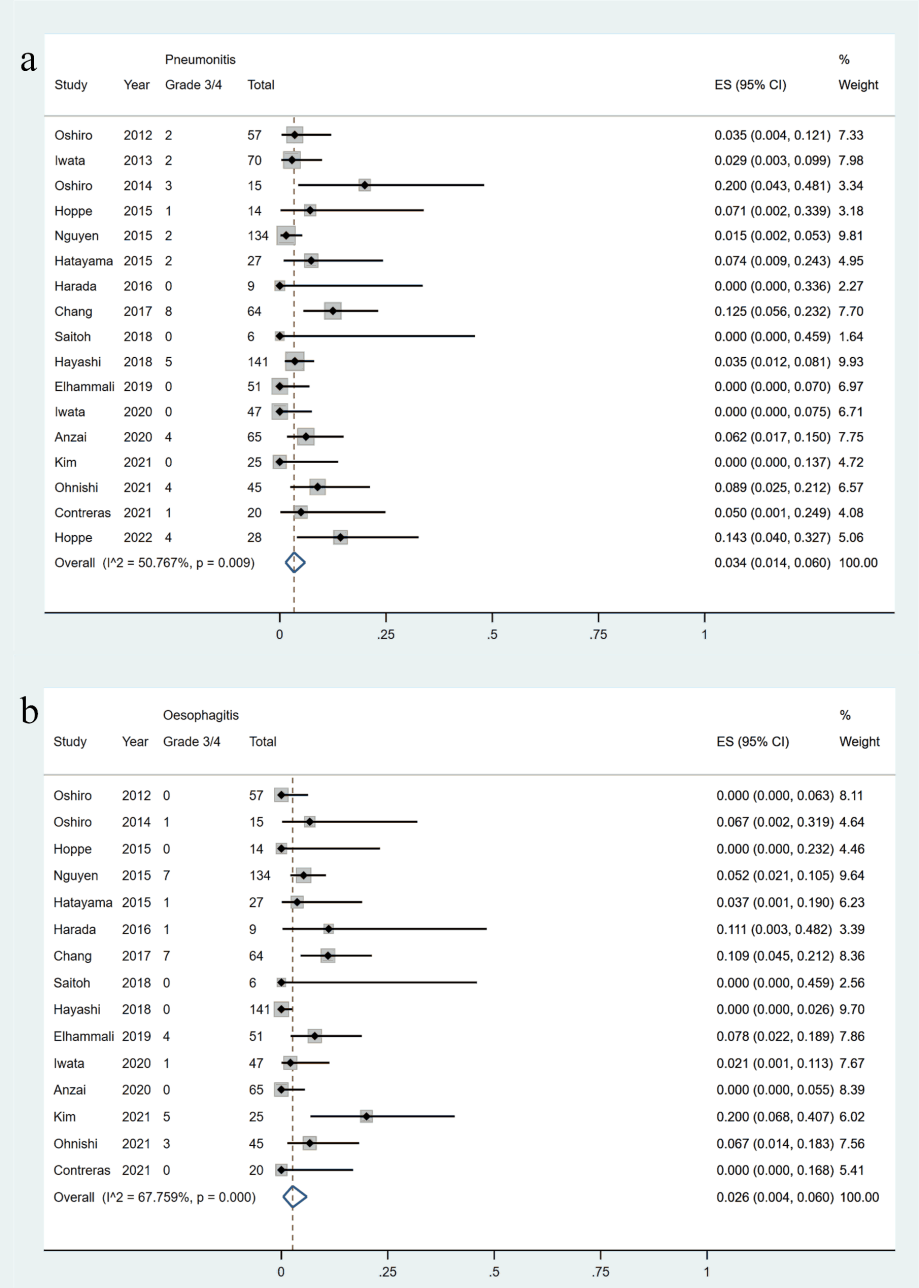
Forest plots of the incidences of grade 3/4 pneumonitis (a) and oesophagitis (b)

Regarding grade 2 pneumonitis, the incidence was 13.1%, and the PBT and CCRT groups had similar incidences (14.6% vs. 14.7%), both higher than that in the CIRT group (6.5%) (Supplementary Fig. S6a-b). The incidences of grade 2 pneumonitis did not vary significantly among subgroups stratified by stage (Supplementary Fig. S6c). The incidence of grade 3/4 pneumonitis was low, at 3.4% (95%CI = 1.4-6.0%), and the incidences were similar among the subgroups stratified by treatment type or stage (Fig. 5a and Supplementary Fig. S7a-b).

The incidences of grade 2 and grade 3/4 oesophagitis were 23.2% (95%CI = 13.7-34.2%), 2.6% (95%CI = 0.4-6.0%), respectively (Supplementary Fig. S8a and Fig. 5b). The results of treatment type subgroup analysis showed that the incidences of grade 2/3/4 esophagitis was found to be significantly lower in patients treated with PBT or CIRT as compared to those who received CCRT. The incidences of grade 2 esophagitis in PBT, CIRT, and CCRT groups were 6.0%, 2.3%, and 33.1%, respectively (Supplementary Fig. S8b). The incidences of grade 3/4 esophagitis in PBT, CIRT, and CCRT groups were 0.4%, 0.0%, and 5.5%, respectively (Supplementary Fig. S9). There was no difference in the incidences of esophagitis (grade 2/3/4) between the two subgroups stratified by stage (Supplementary Fig. S10a-b).

### Treatment‑related death

Two studies reported treatment-related deaths [32, 43]. Two patients who received PBT without concurrent chemotherapy and one who received PBT with concurrent chemotherapy encountered grade 5 adverse events. One patient who had preexisting severe inflammatory pneumonia before the diagnosis of NSCLC died of pneumonia during treatment. Another patient, who had undergone repeated biopsies of the irradiated bronchus, died of hemoptysis after treatment. In addition, One patient with tumor invasion of the pulmonary artery died of bronchopulmonary hemorrhage after treatment.

### Sensitivity analysis

In the analysis of complete cases, the confidence intervals were found to overlap with those of the primary analysis, and the pooled estimate remained in close proximity to the overall estimate. The complete-case analysis shows the pooled 2-year OS, PFS, and LC rates were 58.9% (95% CI = 50.6-68.6%), 38.6% (95% CI = 33.4-44.7%), and 76.2% (95% CI = 68.6-84.6%), respectively (Supplementary Fig. S11-13). Similar outcomes were obtained when each cohort was excluded one at a time (Supplementary Fig. S14-16). The results of the sensitivity analysis indicated that the pooled estimate was reliable.

## Discussion

As far as we know, this is the first meta-analysis that pools the effectiveness of particle therapy in the treatment of LA-NSCLC. The findings indicate that particle therapy is an effective therapeutic schedule for LA-NSCLC (median OS range from 21.3 to 49.1 months and median PFS range from 10.2 to 18.0 months), with acceptable side effects (the incidence of side effects above grade 3 was less than 4%).

Local recurrence or metastasis has been a persistent problem faced by LA-NSCLC patients. For a long time in the past, the standard treatment regimen for inoperable stage III NSCLC was concurrent chemoradiotherapy, but its efficacy was not satisfactory, and doctors constantly strived to seek a breakthrough. Despite the potential benefits of systemic therapies such as targeted therapy and immunotherapy in reducing local recurrence and metastasis, it is undeniable that intensification of radiation remains the most effective approach to address this issue.

Early reports showed that concurrent chemoradiation with dose enhancement seemed to improve the local control rate and transform the pet into a better overall survival rate. A pooled analysis of seven Radiation Therapy Oncology Group studies involving 1356 patients with LA-NSCLC who received chemoradiation indicated that each 1 Gy biologically effective dose (BED) increase in radiation dose was associated with a 4% improvement in survival rate and a 3% increase in local control [11]. The RTOG0617 trial has tried to achieve better survival benefits by increasing the dose of radiation therapy. However, the results showed that patients treated with a high-dose radiation regimen (37 × 2 Gy) exhibited a higher rate of local failure (38.6%) compared to those receiving a conventional radiation regimen (30 × 2 Gy) (30.7%). Median OS and PFS (20.3 months, 9.8 months) were shorter than the conventional dose (28.7 months, 11.8 months). The OS and PFS rates at two years were 57.6%, 29.1% (standard-dose group), and 44.6%, 21.4% (high-dose group), respectively. The 5-year OS and PFS rates were 32.1%, 18.3% (standard-dose group), and 23.0%, 13.0% (high-dose group), respectively [5, 12]. An independent prognostic factor for (OS) in this trial was the mean dose to the heart, which may have contributed to the poorer prognosis in the high radiation dose arm. A study involving 711 patients with non-metastatic NSCLC who received definitive radiotherapy revealed that a greater low-dose bath, particularly lung V5, was linked to lymphopenia, which was a significant prognostic factor for both overall survival and event-free survival (EFS) [47]. As the immune response appears to play a key role in the tumor evolution of NSCLC [48], we can hypothesize that minimizing lung volume exposure can improve clinical outcomes. Similar findings have also been reported by Speirs et al., in that OS in NSCLC patients receiving photon-based chemoradiotherapy was independently correlated with heart V50 and lung V5. [49]. By exploiting the Bragg peak dose distribution, particle therapy could allow higher doses to be delivered to the tumor area without causing excessive dose to the surrounding normal organs, including the heart and lungs, as demonstrated in several retrospective studies [50, 51]. This theory could also be proved by several studies on the treatment of LA-NSCLC with PBT (37 × 2 GyE) combined with concurrent chemotherapy, which had achieved relatively high 2-year or 5-year OS [34, 37, 41].

In addition, the researchers found that when the total duration of photon radiotherapy exceeded six weeks (30 fractions), each additional day of treatment resulted in a 1.6% decline in survival rate, potentially due to accelerated repopulation of clonogenic tumor cells during the treatment process [52, 53]. Modified accelerated radiotherapy has been reported as more effective than conventionally fractionated radiotherapy in counteracting the time factor-associated loss associated with conventionally fractionated radiotherapy. By reducing the treatment time and increasing the biologically effective dose, modified accelerated radiotherapy may have decreased the accelerated tumor repopulation [54, 55]. In a series of studies on photon radiation therapy, researchers found that the OS rate may be improved to some extent by hypofractionated radiotherapy, but a higher risk of toxicity is also conferred by it [56–58]. In view of the advantage of dose distribution of particle therapy, hypofractionated radiotherapy seems to be more suitable to perform in particle therapy, and most of the studies included in this meta-analysis are of this kind [25, 31–33, 42, 46]. The 2-year OS rate was 61.3%, and the 2-year PFS rate was 37.9% in the present study. Additionally, the 5-year OS rate was 41.3%, and the 5-year PFS rate was 25.3%. The result of the PACIFIC Trial showed the estimated 5-year OS and PFS of patients with stage III NSCLC who received consolidation therapy with durvalumab after concurrent chemoradiotherapy were 42.9% (38.2-47.4%) and 33.1% (28.0-38.2%) [8, 59, 60]. Given that the Pacific trial demonstrated a significant benefit of duruzumab consolidation therapy after concurrent chemoradiotherapy, the consolidation treatment of immunocheckpoint inhibitors after proton therapy might be more promising.

We performed a subgroup analysis to assess the impact of tumor stage and particle therapy type on the prognosis. Tumor stage is an important prognostic factor of NSCLC [61–63], but the subgroup analysis of this study showed that compared with the stage III group, the stage II-III group does not have a longer overall survival rate as expected. This may be due to the relatively small number of patients with stage II in the study. There were three different treatment types in the studies included in this meta-analysis: PBT, CIRT, and CCRT (PBT combined with concurrent chemotherapy). As shown in the subgroup analysis, the CCRT group had better survival benefits than the patients receiving PBT or CIRT only. A radiotherapy-enhancing effect on tumor volume can be produced by concurrent chemoradiotherapy, thus improving local tumor control, which was also an essential reason for the improvement of patient survival rate [64].

Previous studies have shown that patients who were considered operable but refused surgery had a more favorable prognosis than those who were medically inoperable [21, 65, 66]. However, in this meta-analysis, about half of the included studies did not report the proportion of patients who were medically inoperable. According to the obtained data, the proportion of inoperable patients in medicine in most studies was quite high. Therefore, we believe that this study did not exaggerate the effectiveness of particle therapy because of this factor.

A recent meta-analysis reported the incidences of grade 3 or higher pneumonitis and esophagitis after concurrent chemoradiotherapy in stage III NSCLC was 7.8% and 16.6%, respectively [67], which was similar to that reported in the RTOG 0617 trial (7% and 7% in standard-dose group, 4% and 21% in high-dose group) [12]. The secondary analysis of RTOG 0617 demonstrated that the occurrence rate of pneumonitis in the IMRT (Intensity-modulated radiation) group (3.5%) was lower compared with the 3D-RT group (7.9%) in LA-NSCLC patients receiving concurrent chemoradiotherapy (P = 0.039). The incidences of esophagitis were similar in the two groups (IMRT group 13.2% VS 3D-RT group 15.4%) [68]. In general, the present study found that the incidences of pneumonitis and esophagitis in LA-NSCLC patients after particle therapy were relatively low, 3.4% and 2.6%, respectively, which may benefit from its physical advantages. Based on subgroup analysis, the occurrence rate of esophagitis in the CCRT group was higher than that in the PBT or CIRT group (5.5% in the CCRT group, 0.4% in the PBT group, 0% in the CIRT group, P = 0.000), which may be attributed to concurrent chemotherapy, while the incidences of pneumonitis did not differ among groups (2.3% in CIRT group, 3.6% in PBT group, 3.8% in CCRT group, P = 0.937).

It should be acknowledged that this meta-analysis has several limitations: (1) there were no randomized controlled trials; (2) the fractionated dose schemes differ from study to study; (3) some of the studies adopted the treatment scheme of particle therapy combined with concurrent chemotherapy, while the rest were not, and the regimens of concurrent chemotherapy are not uniform; (4) among included trials, significant potential heterogeneity was detected, which could not be fully explained by subgroup analysis; (5) due to the fact that this study based on published articles and unpublished data was not evaluated, publication bias was inevitable. The results of prospective studies by multiple institutions, such as RTOG 1308, are needed to solve the problem of whether particle therapy can really improve the survival rate of LA-NSCLC patients by reducing the dose to normal organs.

In conclusion, this meta-analysis comprising 19 studies demonstrated that particle therapy might confer promising survival outcomes and acceptable toxicity in LA-NSCLC patients. However, the advantages of particle therapy in comparison to photon therapy require confirmation through additional large-scale, multi-institutional prospective studies.

## Electronic supplementary material

Below is the link to the electronic supplementary material.

Supplementary Material 1

## Abbreviations

CIRT: Carbon-ion radiotherapy
PBT: Proton beam therapy
NSCLC: Non-small cell lung cancer
LA-NSCLC: Locally advanced non-small cell lung cancer
RTOG: Radiation Therapy Oncology Group
PROSPERO: Prospective register of systematic reviews
OS: Overall survival
PFS: Progression-free survival
LC: Local control
BED: Biologically effective dose
IMRT: Intensity-modulated radiation
