# Supplementary material for: Efficacy and safety of particle therapy for inoperable stage II-III non-small cell lung cancer: a systematic review and meta-analysis
Source: Radiat Oncol. 2023 May 22;18:86. doi: 10.1186/s13014-023-02264-x (PMC10201735; doi:10.1186/s13014-023-02264-x)
Supplement: Supplementary file 1 — Supplementary Material 1 [file 13014_2023_2264_MOESM1_ESM.pdf]

Supplement 1. Detailed search strategy.

PubMed

(("Carcinoma, Non-Small-Cell Lung"[Mesh]) OR ((((((Non-Small-Cell Lung Carcinomas[Title/Abstract]) OR (Non-Small-Cell Lung Carcinoma[Title/Abstract])) OR (Non Small Cell Lung Carcinoma[Title/Abstract])) OR (Non-Small Cell Lung Carcinoma[Title/Abstract])) OR (Non-Small Cell Lung Cancer[Title/Abstract])) OR (Nonsmall Cell Lung Cancer[Title/Abstract]))) AND (((((("Heavy Ion Radiotherapy"[Mesh]) OR ("Heavy Ions"[Mesh])) OR ("Proton Therapy"[Mesh])) OR ("Protons"[Mesh])) OR (((proton therap*[Title/Abstract]) OR (proton[Title/Abstract])) AND ((((((beam radiation[Title/Abstract]) OR (radiation[Title/Abstract])) OR (beam irradiation[Title/Abstract])) OR (irradiation[Title/Abstract])) OR (radiotherap*[Title/Abstract])) OR (beam therap*[Title/Abstract])))) OR ((((particle[Title/Abstract]) OR (heavy ion*[Title/Abstract])) OR (carbon[Title/Abstract])) AND (((((((beam radiation[Title/Abstract]) OR (radiation[Title/Abstract])) OR (beam irradiation[Title/Abstract])) OR (irradiation[Title/Abstract])) OR (radiotherap*[Title/Abstract])) OR (beam therap*[Title/Abstract])) OR (ion therap*[Title/Abstract]))))

Embase

#1. 'non small cell lung cancer'/exp

#2. 'non small cell lung carcinomas':ti,ab,kw OR 'non small cell lung cancer':ti,ab,kw OR 'non small cell lung carcinoma':ti,ab,kw OR nsclc:ti,ab,kw

#3. #1 OR #2

#4. 'particle therapy'/exp

#5. 'heavy ion'/exp

#6. 'particle radiation'/exp

#7. 'proton'/exp

#8. 'proton therap*':ti,ab,kw OR proton:ti,ab,kw

#9. particle:ti,ab,kw OR 'heavy ion*':ti,ab,kw OR carbon:ti,ab,kw

#10. 'beam radiation':ti,ab,kw OR radiation:ti,ab,kw OR 'beam irradiation':ti,ab,kw OR irradiation:ti,ab,kw OR radiotherap*:ti,ab,kw OR 'beam therap*':ti,ab,kw

11. 'beam radiation':ti,ab,kw OR radiation:ti,ab,kw OR 'beam irradiation':ti,ab,kw OR

irradiation:ti,ab,kw OR radiotherap*:ti,ab,kw OR 'beam therap*':ti,ab,kw OR 'ion therap*':ti,ab,kw

#12. #8 AND #10

#13. #9 AND #11

#14. #4 OR #5 OR #6 OR #7 OR #12 OR #13

#15. #3 AND #14

Cochrane Library

#1 MeSH descriptor: [Heavy Ion Radiotherapy] explode all trees

#2 MeSH descriptor: [Heavy Ions] explode all trees

#3 MeSH descriptor: [Proton Therapy] explode all trees

#4 MeSH descriptor: [Protons] explode all trees

#5 (proton therap*):ti,ab,kw OR (proton):ti,ab,kw (Word variations have been searched)

#6 (particle):ti,ab,kw OR (heavy ion*):ti,ab,kw OR (carbon):ti,ab,kw (Word variations have been searched)

#7 (beam radiation):ti,ab,kw OR (radiation):ti,ab,kw OR (beam irradiation):ti,ab,kw OR (irradiation):ti,ab,kw OR (radiotherap*):ti,ab,kw (Word variations have been searched)

#8 (beam therap*):ti,ab,kw (Word variations have been searched)

#9 (ion therap*):ti,ab,kw (Word variations have been searched)

#10 #7 OR #8

#11 #7 OR #8 OR #9

#12 #5 AND #10

#13 #6 AND #11

#14 #1 OR #2 OR #3 OR #4 OR #12 OR #13

#15 MeSH descriptor: [Carcinoma, Non-Small-Cell Lung] explode all trees

#16 (Non Small Cell Lung Carcinoma):ti,ab,kw OR (Non-Small-Cell Lung Carcinoma):ti,ab,kw OR (Carcinomas, Non-Small-Cell Lung):ti,ab,kw OR (Non-Small-Cell Lung Carcinomas):ti,ab,kw OR (Carcinoma, Non-Small Cell Lung):ti,ab,kw (Word variations have been searched)

#17 (Non-Small Cell Lung Cancer):ti,ab,kw OR (Carcinoma, Non Small Cell Lung):ti,ab,kw OR (Non-Small Cell Lung Carcinoma):ti,ab,kw OR (Lung Carcinoma, Non-Small-Cell):ti,ab,kw OR (Nonsmall Cell Lung Cancer):ti,ab,kw (Word variations have been searched)

#18 (Lung Carcinomas, Non-Small-Cell):ti,ab,kw (Word variations have been searched)

#19 #15 OR #16 OR #17 OR #18

#20 #14 AND #19

Web of science

#1: ((((((TS=("Carcinoma, Non-Small-Cell Lung")) OR TS=("Non-Small-Cell Lung Carcinomas")) OR TS=("Non-Small-Cell Lung Carcinoma")) OR TS=("Non Small Cell Lung Carcinoma")) OR TS=("Non-Small Cell Lung Carcinoma")) OR TS=("Non-Small Cell Lung Cancer")) OR TS=("Nonsmall Cell Lung Cancer")

#2: (((TS=("particle therapy")) OR TS=("particle radiation")) OR TS=("Heavy Ion Radiotherapy")) OR TS=("Proton Therapy")

#3: (TS=("proton therap*")) OR TS=(proton)

#4: ((((TS=("beam radiation")) OR TS=("radiation OR beam irradiation")) OR TS=(irradiation)) OR TS=("radiotherap*")) OR TS=("beam therap*")

#5: #3 AND #4

#6: ((TS=(particle)) OR TS=("heavy ion*")) OR TS=(carbon)

#7: ((((((TS=("beam radiation")) OR TS=(radiation)) OR TS=("beam irradiation")) OR TS=(irradiation)) OR TS=(radiotherap*)) OR TS=("beam therap*")) OR TS=("ion therap*")

#8: #6 AND #7

#9: #2 OR #5 OR #8

#10: #1 AND #9

Supplementary table 1. Quality assessment of eligible studies.

			Methodological item												MINORS score index
Study	Nation	Year	①	②	③	④	⑤	⑥	⑦	⑧	⑨	⑩	⑪	⑫	
Nakayama	Japan	2011	2	2	1	2	1	1	1	0					10
Oshiro	Japan	2012	2	2	2	2	1	1	1	0					11
Iwata	Japan	2013	2	2	1	2	1	2	1	0					11
Oshiro	Japan	2014	2	2	2	2	1	1	1	0					11
Hoppe	America	2015	2	2	1	2	1	2	1	0					11
Nguyen	America	2015	2	2	2	2	1	2	1	0					12
Hatayama	Japan	2015	2	2	2	2	1	1	1	0					11
Harada	Japan	2016	2	2	1	2	1	2	1	0					11
Chang	America	2017	2	2	2	2	1	2	1	0					12
Shirai	Japan	2017	2	2	2	2	1	2	1	0					12
Saitoh	Japan	2018	2	2	2	2	1	2	1	0					12
Hayashi	Japan	2018	2	2	2	2	1	2	1	0					12
Elhammali	America	2019	2	2	2	2	1	1	1	0					11
Iwata	Japan	2020	2	2	1	2	1	2	1	0					11
Anzai	Japan	2020	2	2	2	2	1	2	1	0					12
Kim	Korea	2021	2	2	1	2	1	1	1	0	2	2	2	2	18
Ohnishi	Japan	2021	2	2	1	2	1	2	1	0					11
Contreras	America	2021	2	2	1	2	1	1	1	0					10
Hoppe	America	2022	2	2	1	2	1	2	1	0					11

The items are scored 0 (not reported), 1 (reported but inadequate) or 2 (reported and adequate). The global ideal score being 16 for non-comparative studies and 24 for comparative studies. < 50% Poor, 51–80% fair, > 80% good.

① A clearly stated aim; ② Inclusion of consecutive patients; ③ Prospective collection of data; ④ Endpoints appropriate to the aim of the study; ⑤ Unbiased assessment of the study endpoint; ⑥ Follow-up period appropriate to the aim of the study; ⑦ Loss to follow up less than 5%; ⑧ Prospective calculation of the study size. Additional criteria in the case of comparative study: ⑨ An adequate control group; ⑩ Contemporary groups; ⑪ Baseline equivalence of groups; ⑫ Adequate statistical analyses.

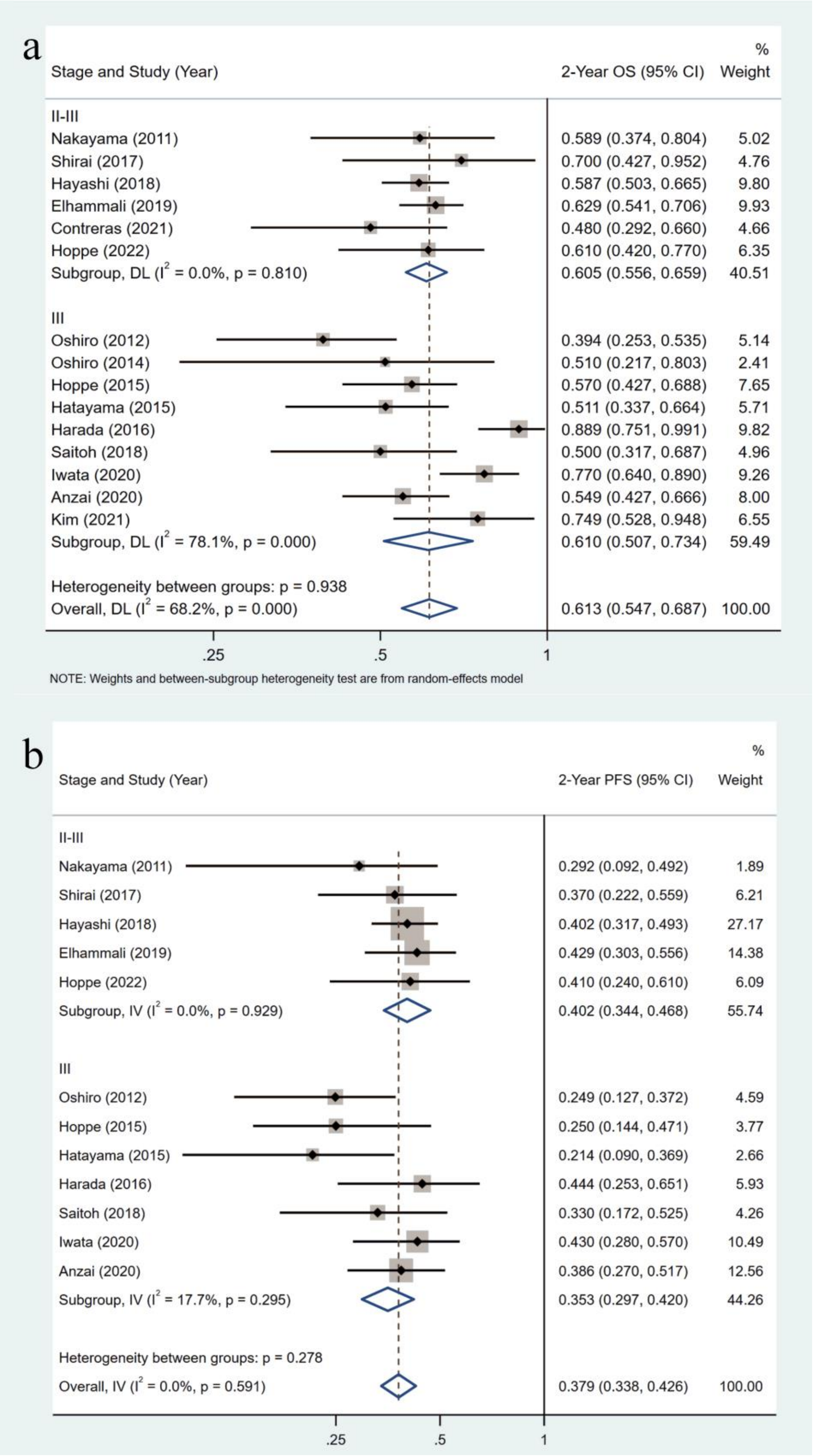

Supplementary Fig. S1. Subgroup analysis of the 2-year overall survival rate (OS) (a) and progression-free survival (PFS) (b) stratified by stage.

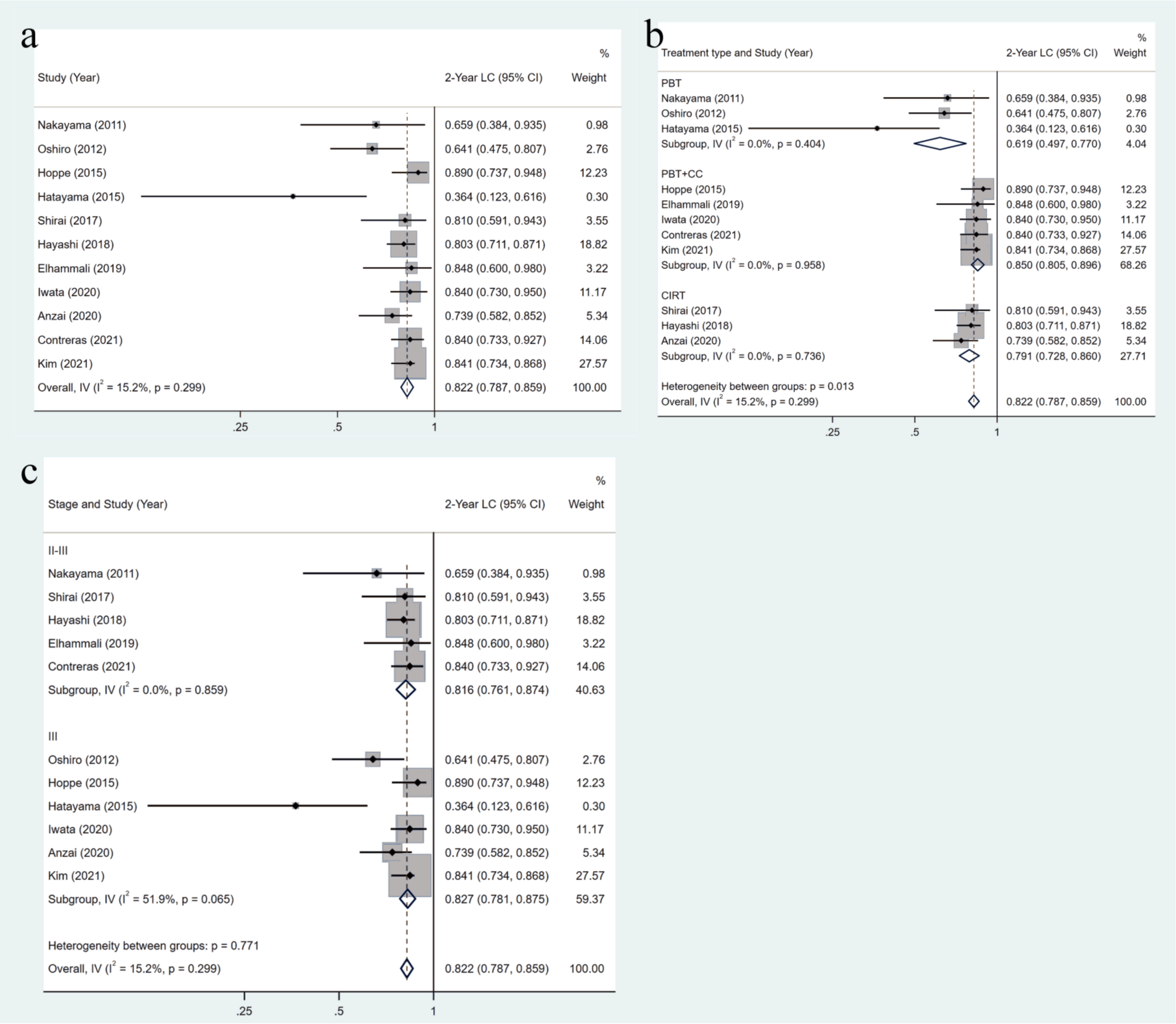

Supplementary Fig. S2. Meta-analysis of the 2-year local control rate (LC): (a) 2-year LC, overall; (b) 2-year LC, subgroup analysis stratified by treatment type; (c) 2-year LC, subgroup analysis stratified by stage. Abbreviations: PBT, proton beam therapy; CIRT, carbon-ion radiotherapy; CC, concurrent chemotherapy.

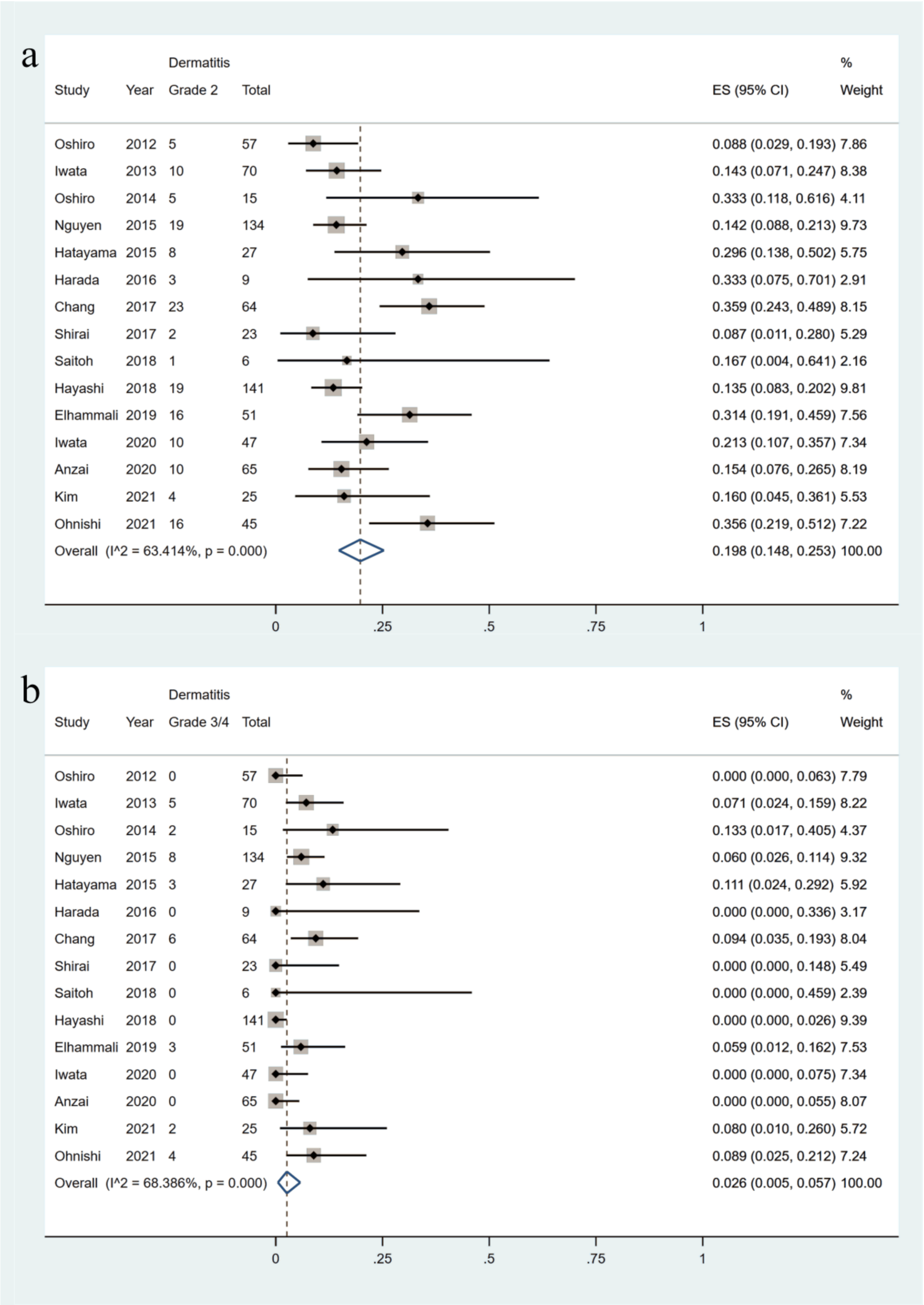

Supplementary Fig. S3. Forest plots of the incidence of dermatitis: (a) incidence of grade 2 dermatitis, overall; (b) incidence of grade 3/4 dermatitis, overall.

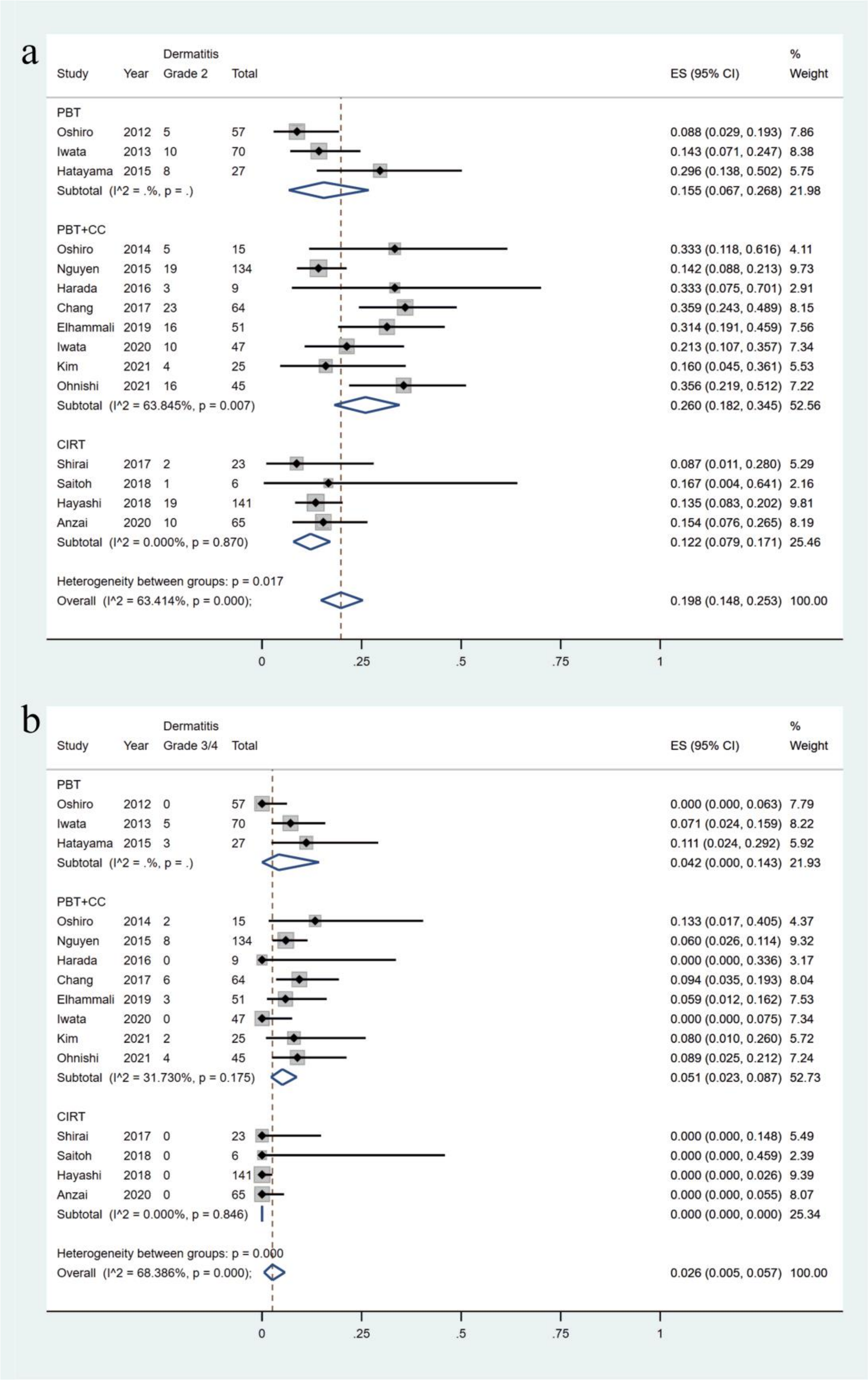

Supplementary Fig. S4. Subgroup analysis of the incidence of grade 2 (a) and grade 3/4 (b) dermatitis stratified by treatment type. Abbreviations: PBT, proton beam therapy; CIRT, carbon-ion radiotherapy; CC, concurrent chemotherapy.

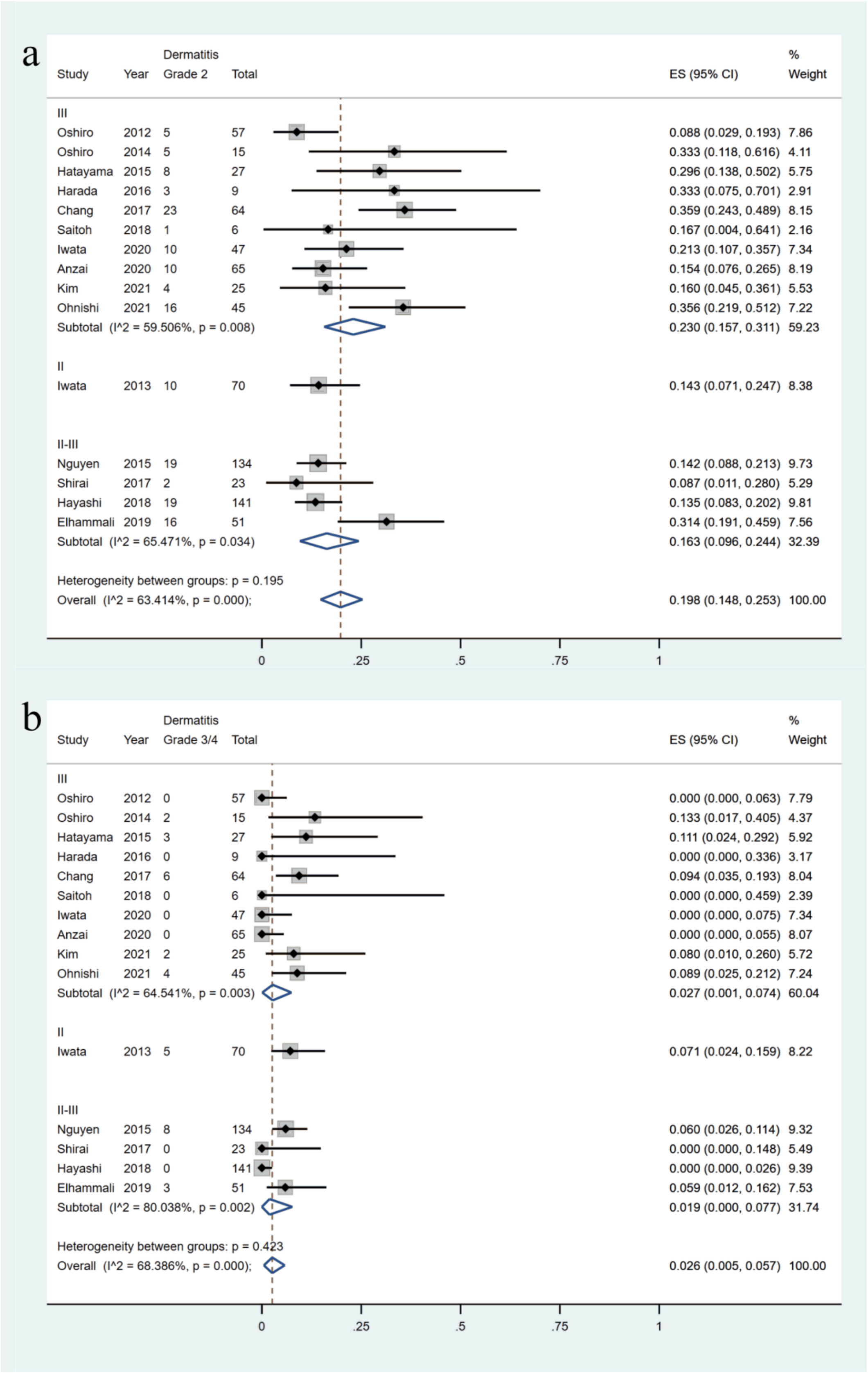

Supplementary Fig. S5. Subgroup analysis of the incidence of grade 2 (a) and grade 3/4 (b) dermatitis stratified by stage.

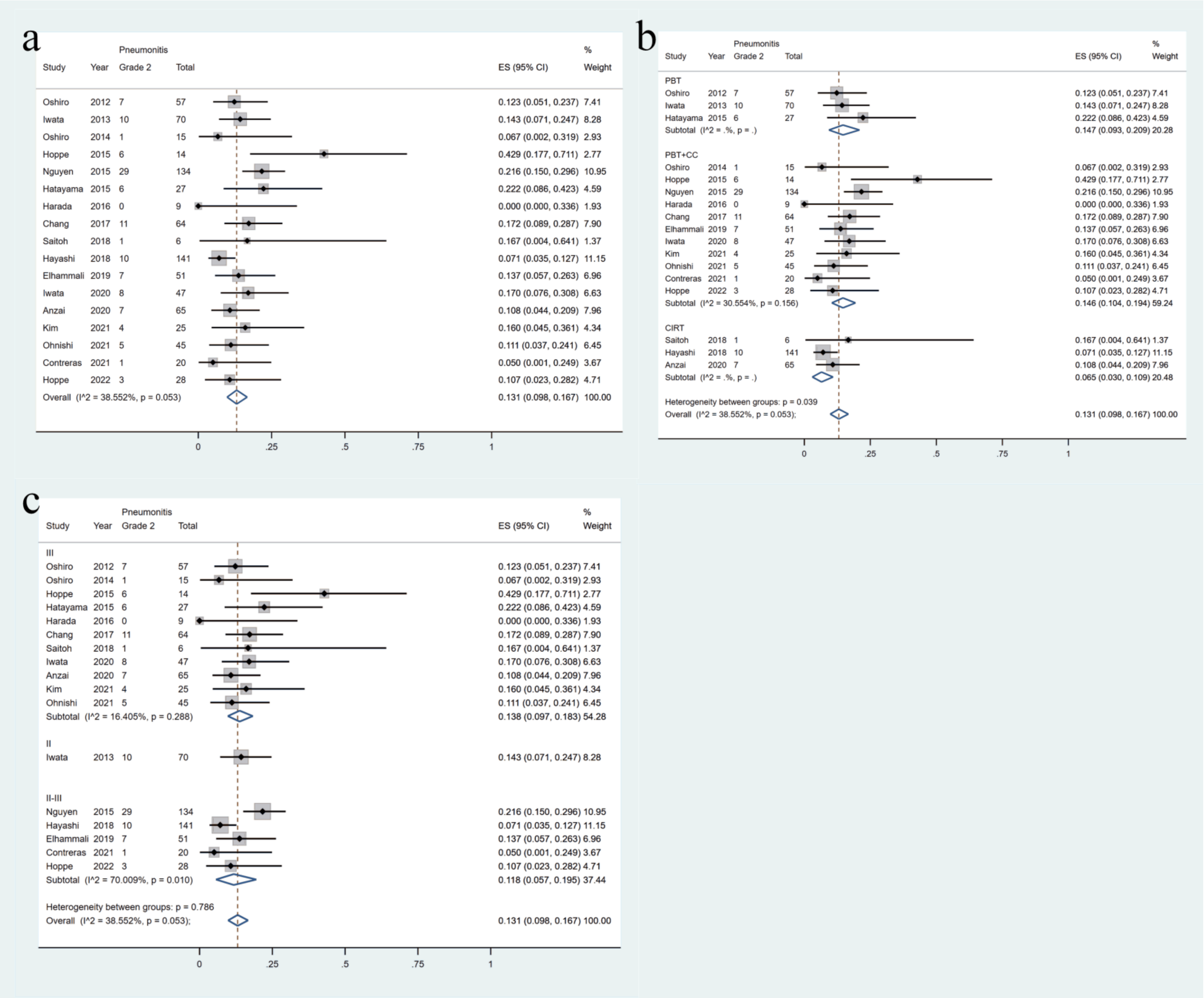

Supplementary Fig. S6. Forest plots of the incidence of grade 2 pneumonitis: (a) incidence of grade 2 pneumonitis, overall; (b) subgroup analysis stratified by treatment type; (c) subgroup analysis stratified by stage. Abbreviations: PBT, proton beam therapy; CIRT, carbon-ion radiotherapy; CC, concurrent chemotherapy.

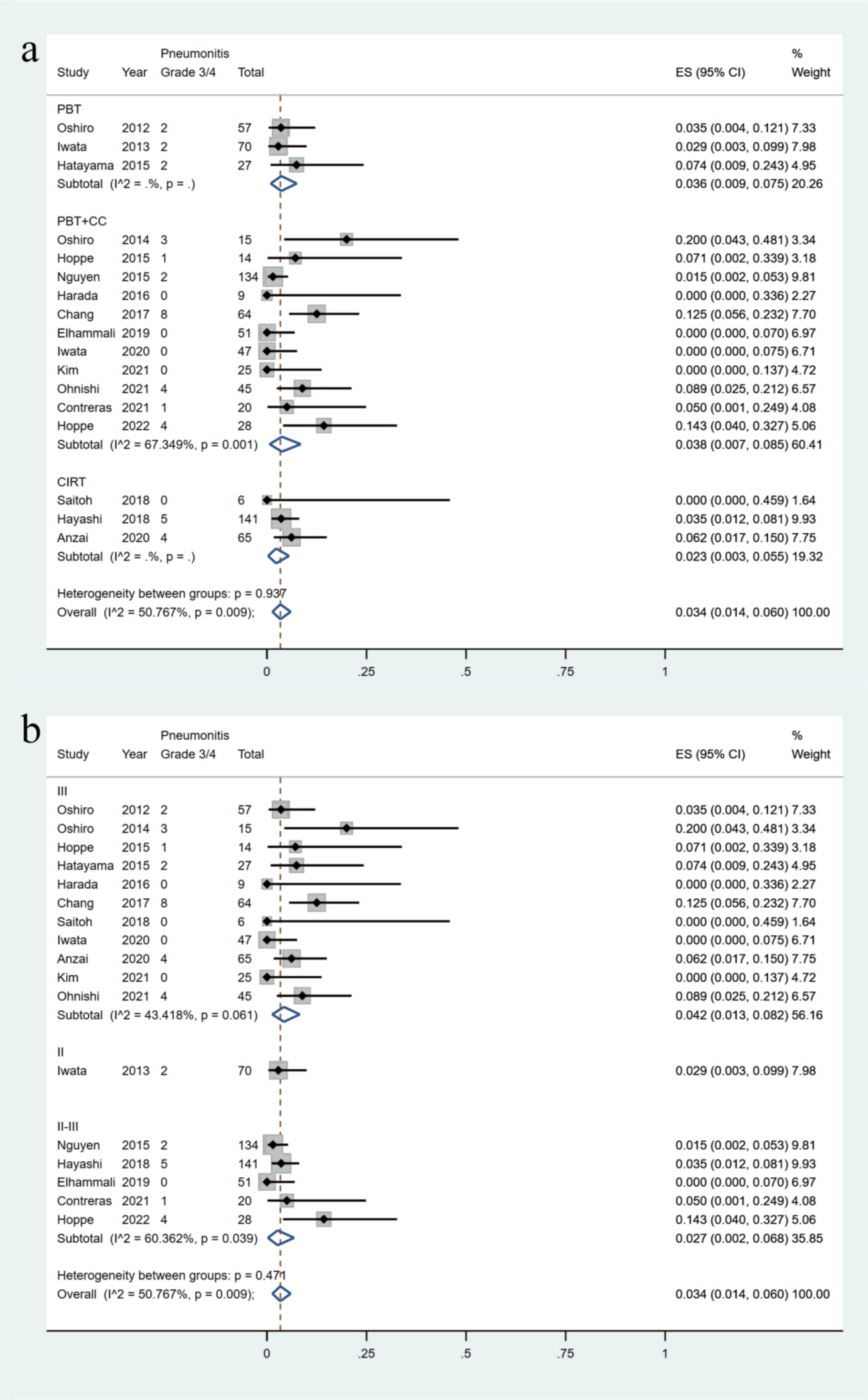

Supplementary Fig. S7. Subgroup analysis of the incidence of grade 3/4 pneumonitis stratified by treatment type (a) and stage (b). Abbreviations: PBT, proton beam therapy; CIRT, carbon-ion radiotherapy; CC, concurrent chemotherapy.

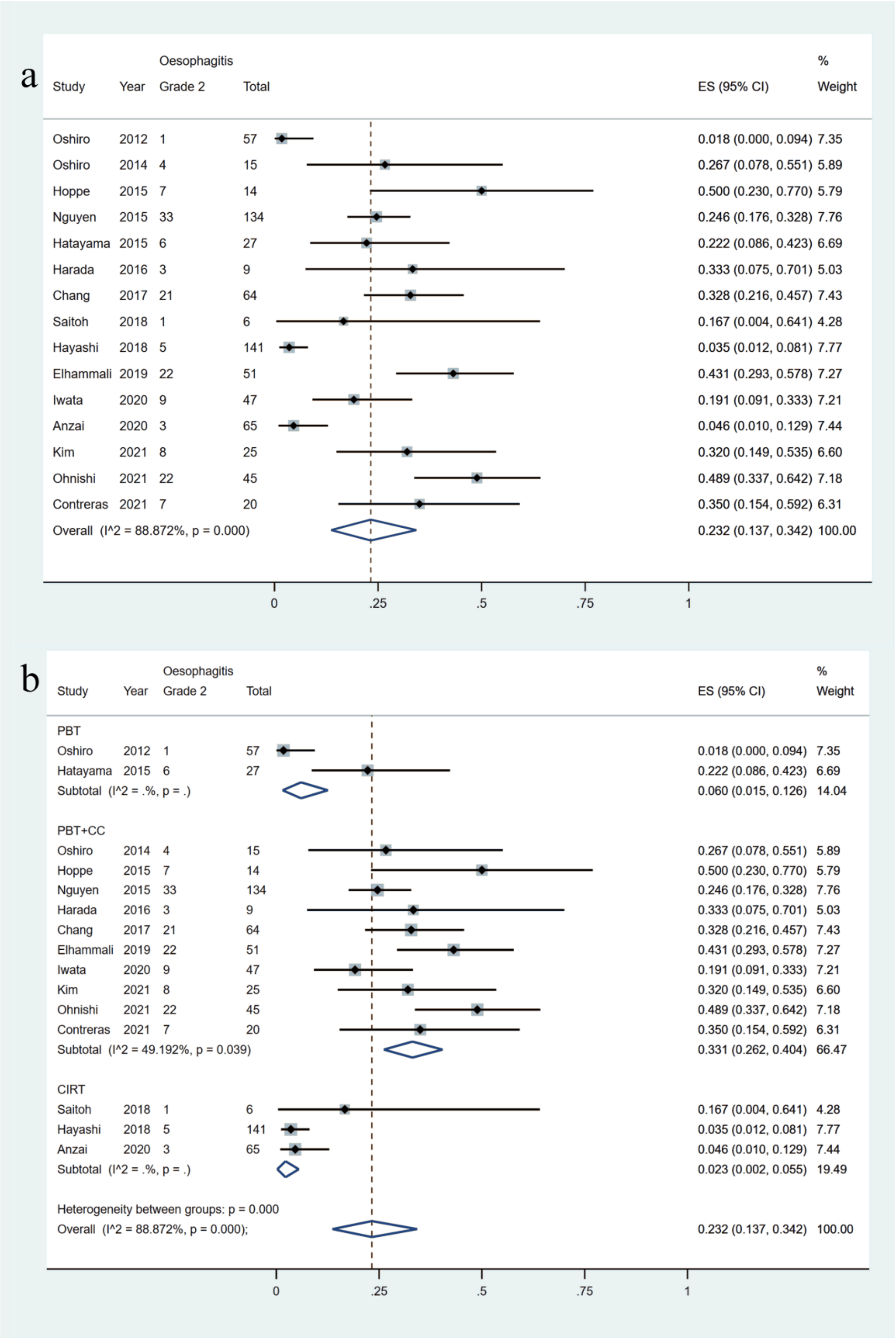

Supplementary Fig. S8. Meta-analysis of the incidence of oesophagitis: (a) incidence of grade 2 oesophagitis, overall; (b) subgroup analysis of grade 2 oesophagitis stratified by treatment type. Abbreviations: PBT, proton beam therapy; CIRT, carbon-ion radiotherapy; CC, concurrent chemotherapy.

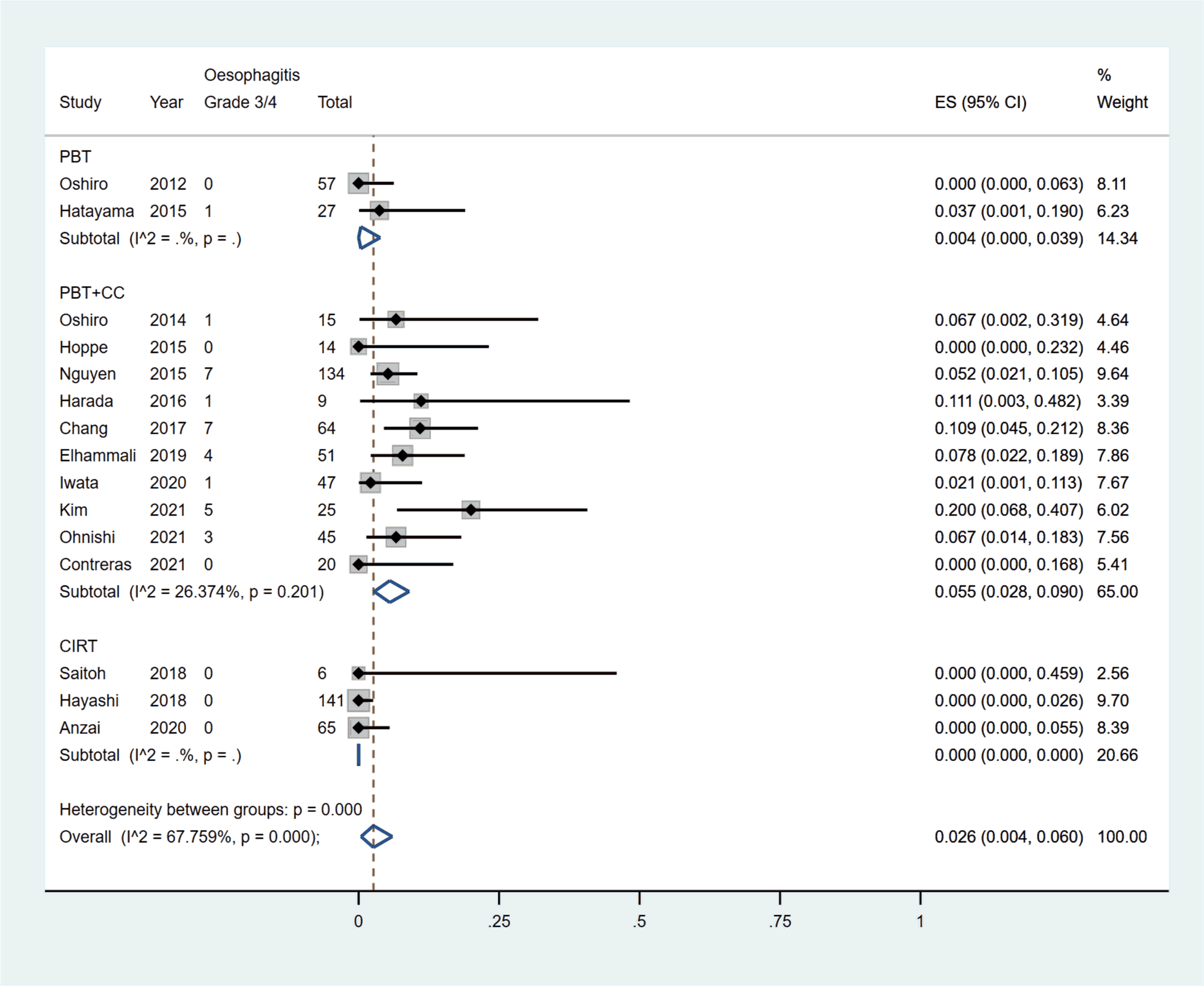

Supplementary Fig. S9. Subgroup analysis of grade 3/4 oesophagitis stratified by treatment type. Abbreviations: PBT, proton beam therapy; CIRT, carbon-ion radiotherapy; CC, concurrent chemotherapy.

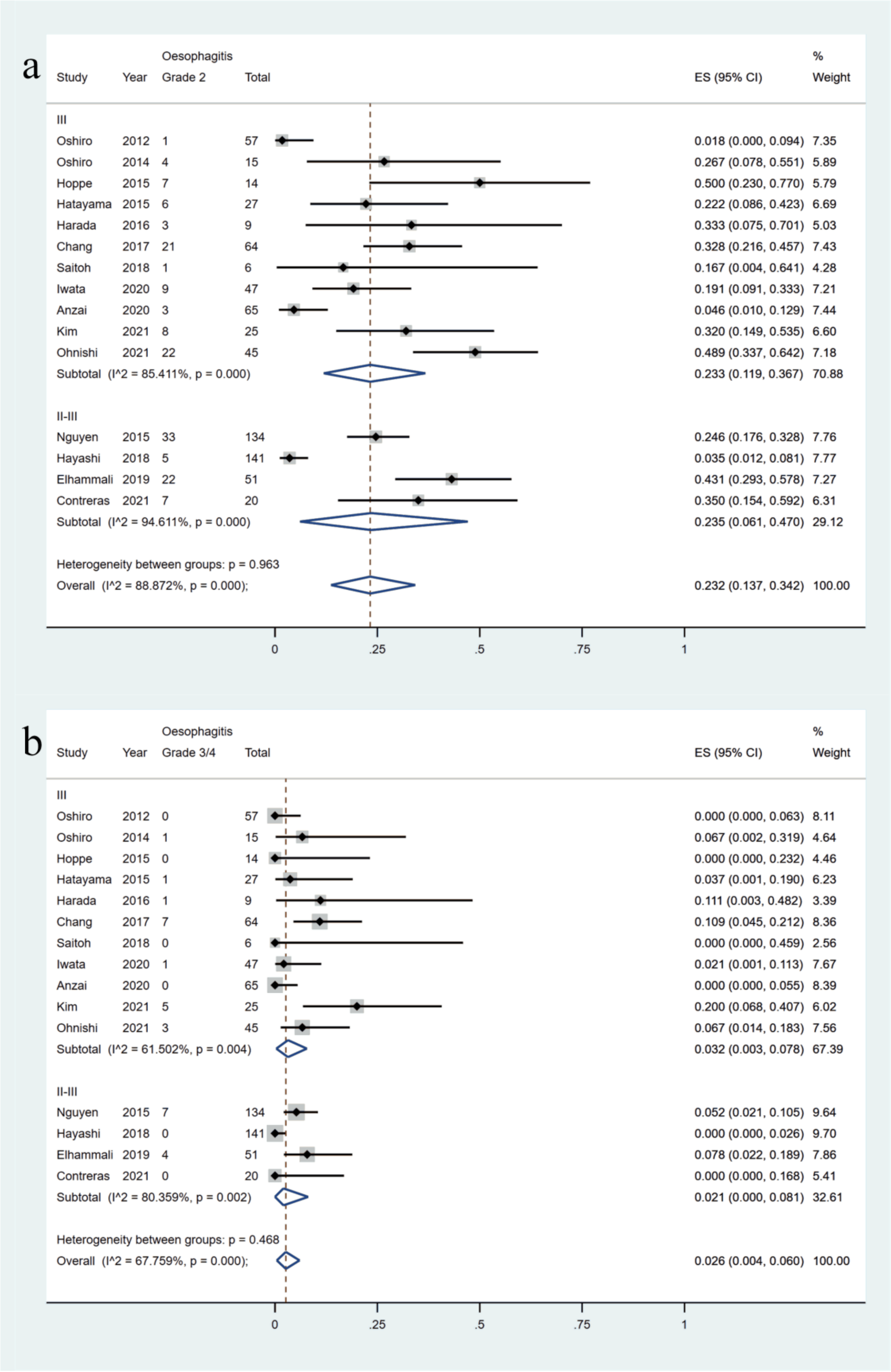

Supplementary Fig. S10. Subgroup analysis of the incidence of grade 2 (a) and grade 3/4 (b) oesophagitis stratified by stage.

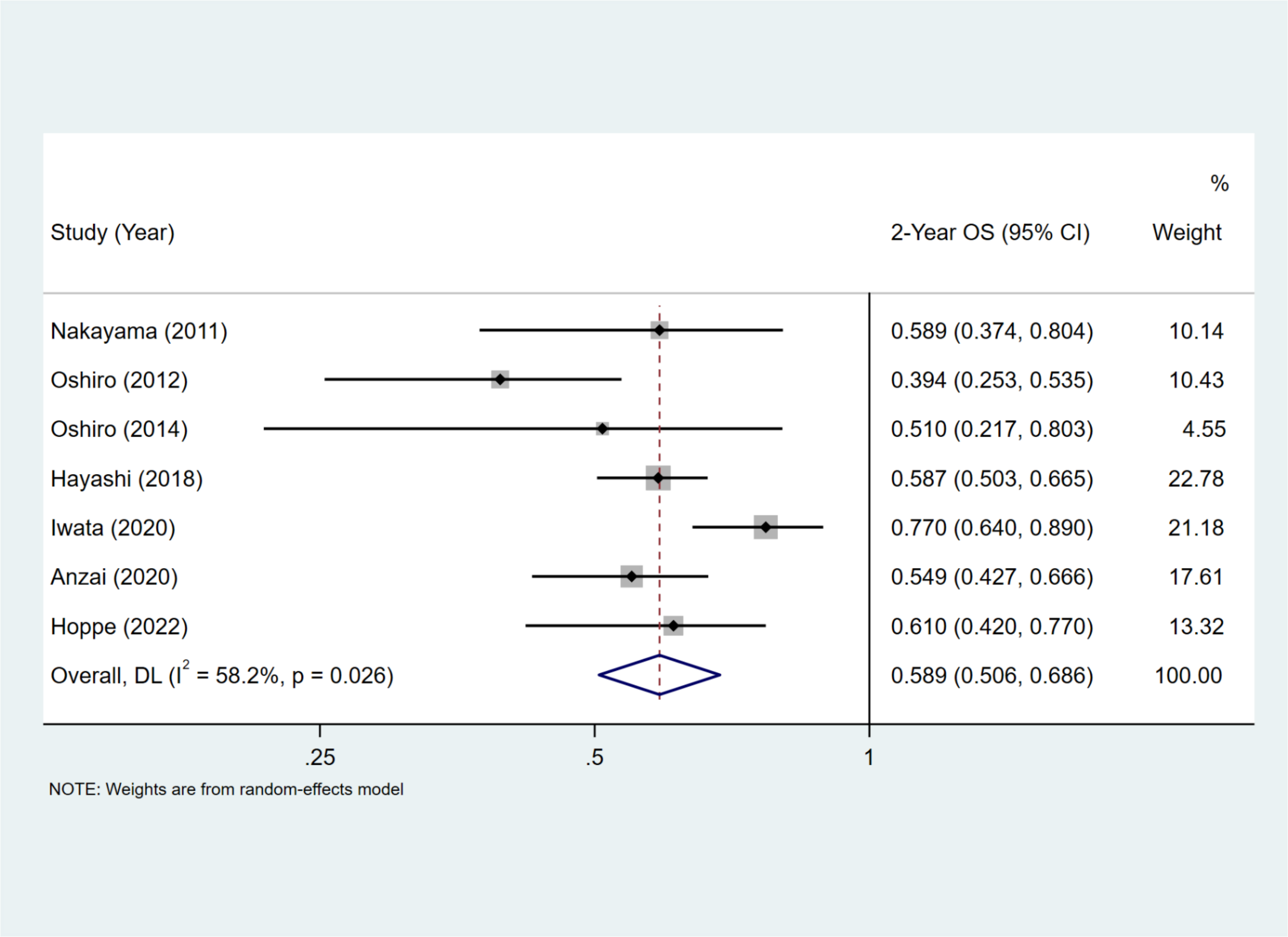

Supplementary Fig. S11. Sensitivity analysis of 2-year overall survival (OS) : complete-case analysis.

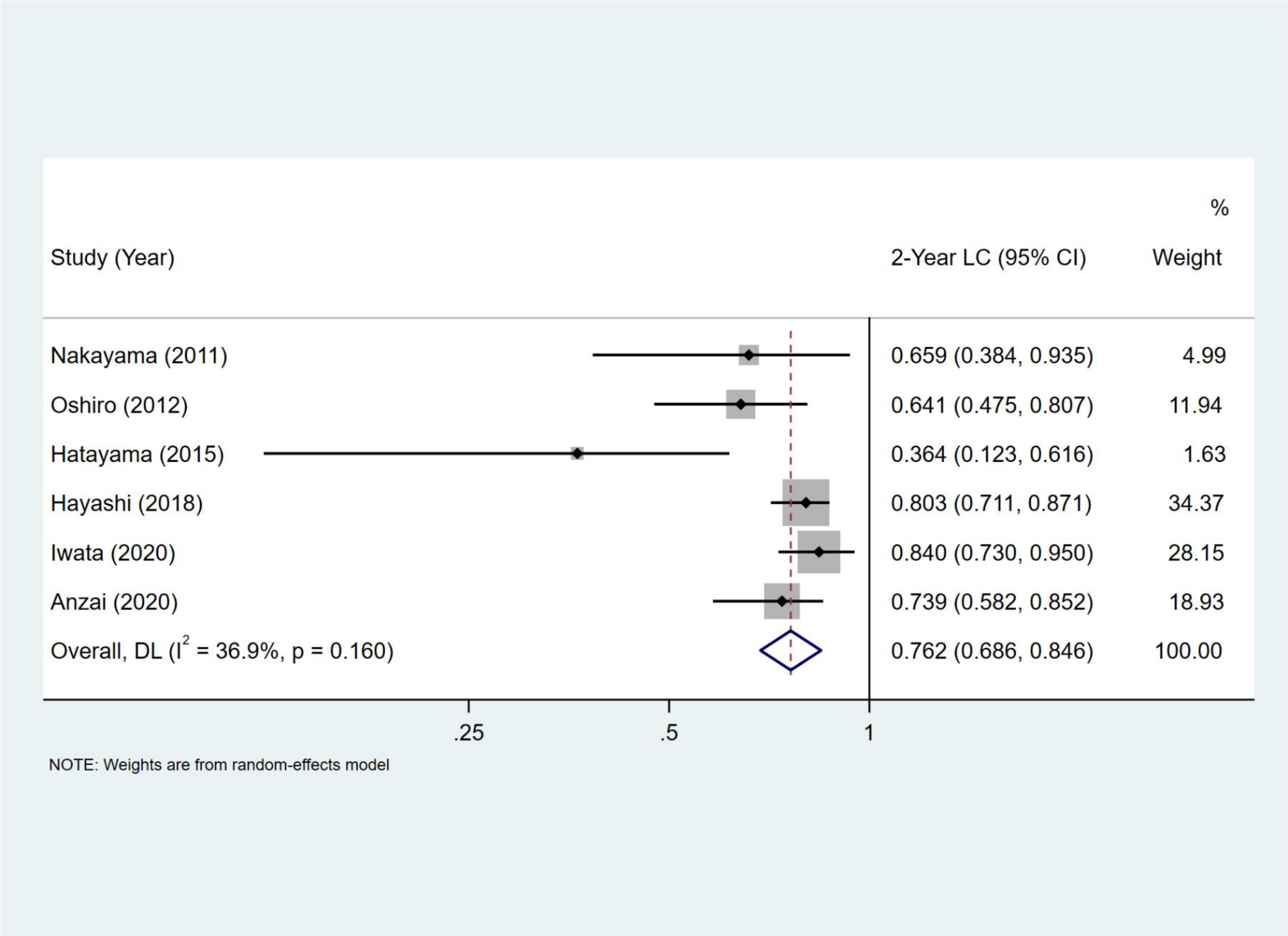

Supplementary Fig. S12. Sensitivity analysis of 2-year progression-free survival (PFS): complete-case analysis.

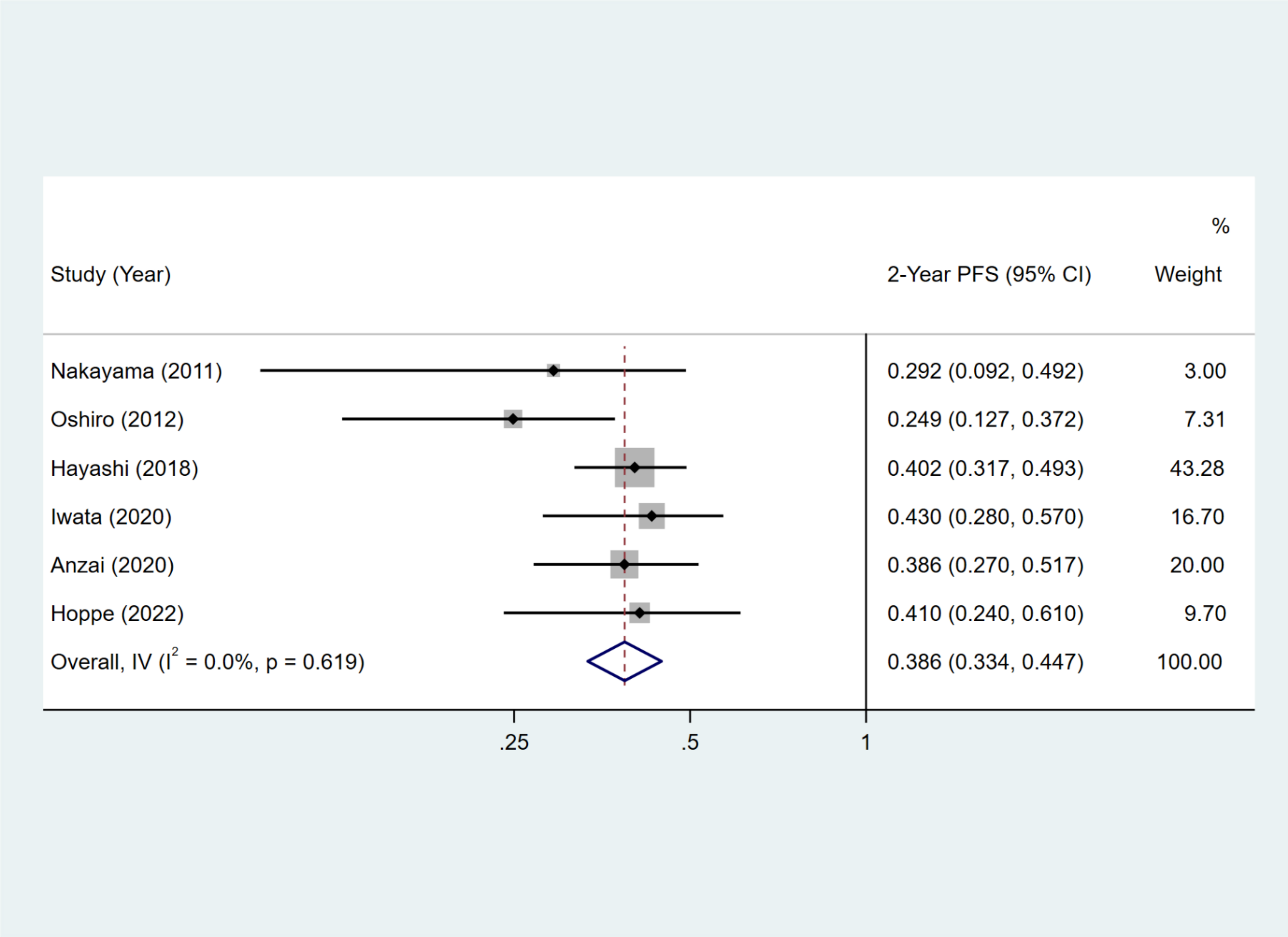

Supplementary Fig. S13. Sensitivity analysis of 2-year local control (LC): complete-case analysis..

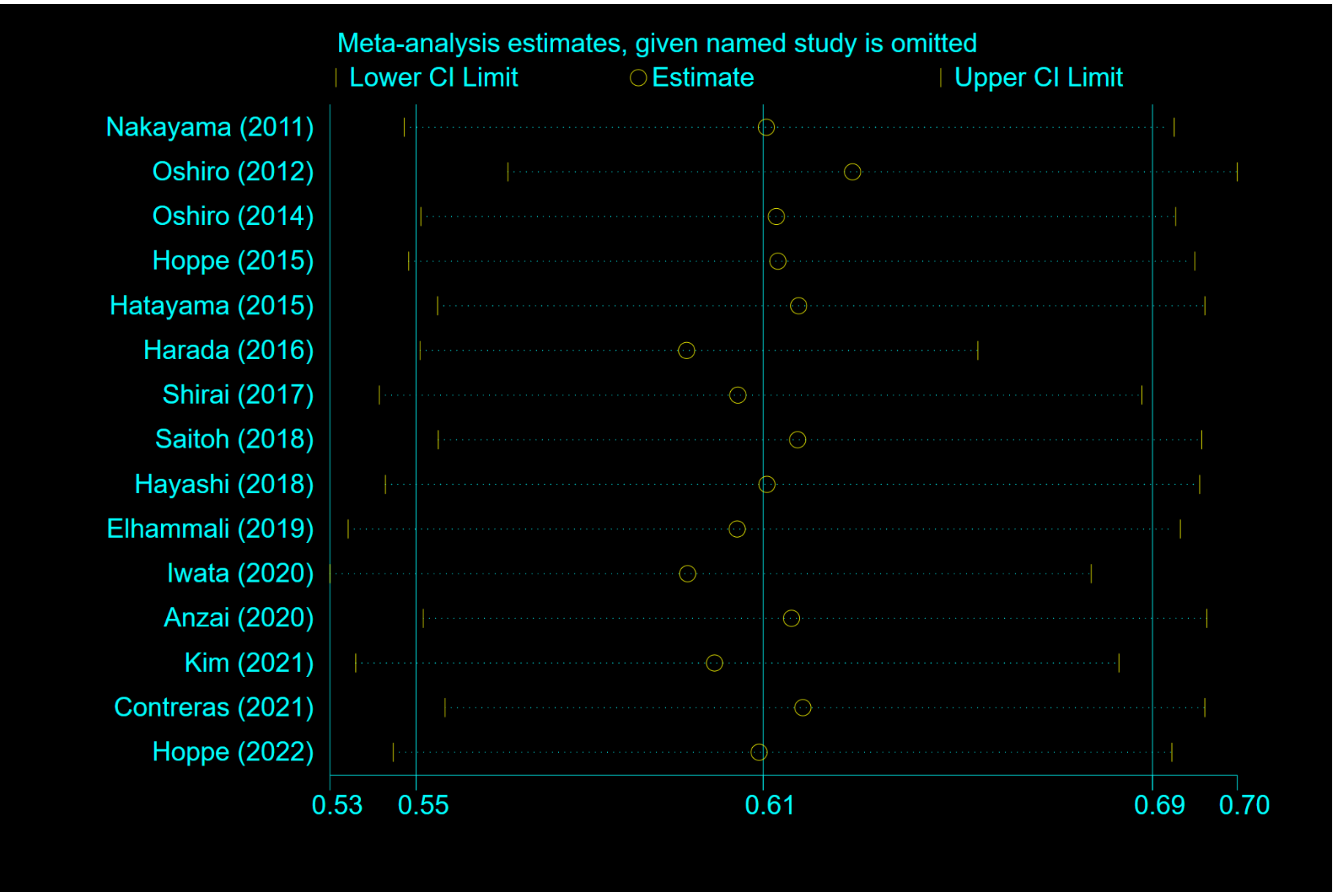

Supplementary Fig. S14. Sensitivity analysis of 2-year overall survival (OS): excluding one cohort at a time.

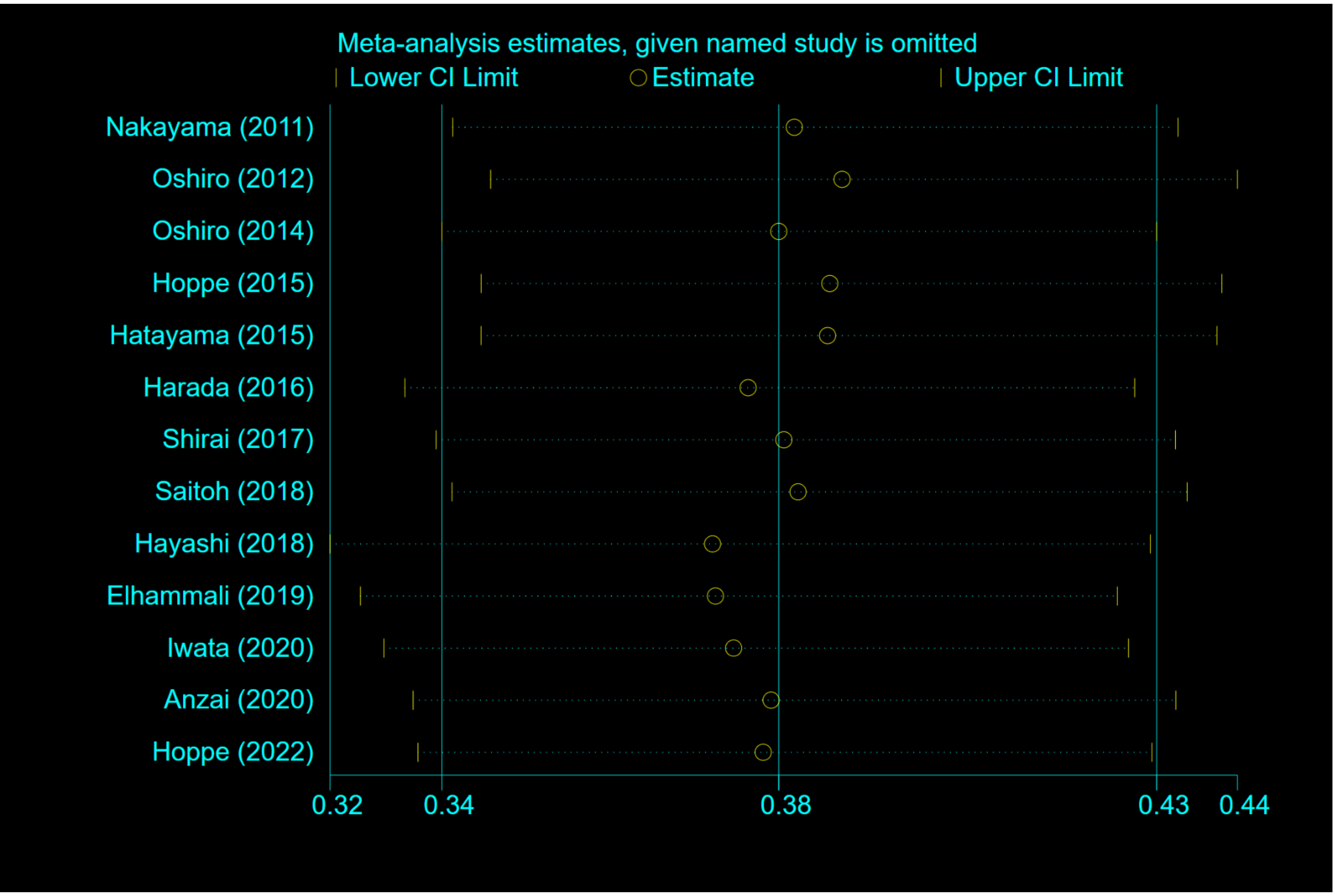

Supplementary Fig. S15. Sensitivity analysis of 2-year progression-free survival (PFS): excluding one cohort at a time.

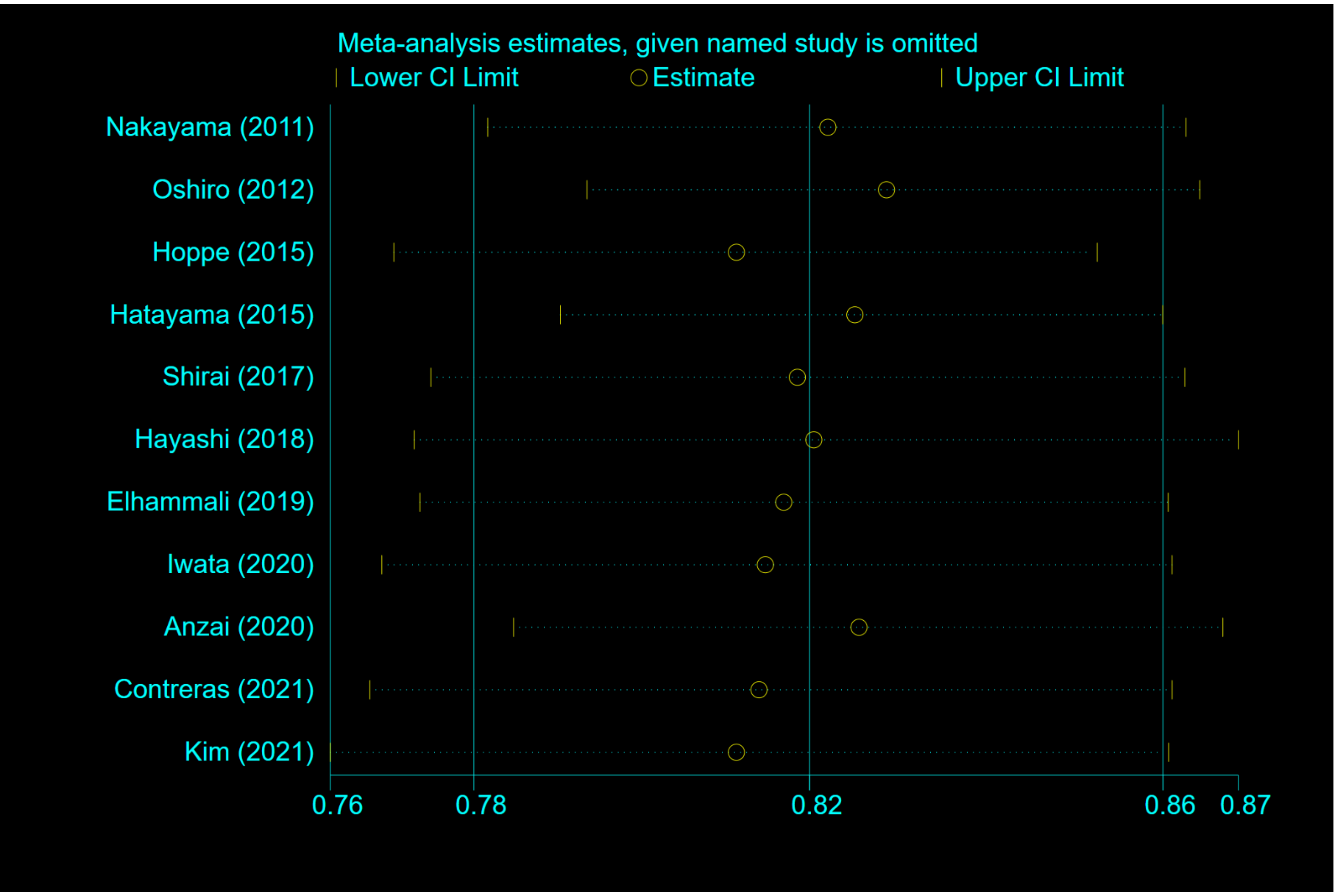

Supplementary Fig. S16. Sensitivity analysis of 2-year local control (LC): excluding one cohort at a time.
